# Simultaneous multislice acquisition with multi-contrast segmented EPI for separation of signal contributions in dynamic contrast-enhanced imaging

**DOI:** 10.1371/journal.pone.0202673

**Published:** 2018-08-28

**Authors:** Klaus Eickel, David Andrew Porter, Anika Söhner, Marc Maaß, Lutz Lüdemann, Matthias Günther

**Affiliations:** 1 University Bremen, Bermen, Germany; 2 Fraunhofer MEVIS, Bremen, Germany; 3 mediri GmbH, Heidelberg, Germany; 4 University of Glasgow, Glasgow, United Kingdom; 5 University Hospital Essen, Essen, Germany; 6 Evangelisches Krankenhaus Wesel, Wesel, Germany; University of California Berkeley, UNITED STATES

## Abstract

We present a method to efficiently separate signal in magnetic resonance imaging (MRI) into a base signal S0, representing the mainly T1-weighted component without T2*-relaxation, and its T2*-weighted counterpart by the rapid acquisition of multiple contrasts for advanced pharmacokinetic modelling. This is achieved by incorporating simultaneous multislice (SMS) imaging into a multi-contrast, segmented echo planar imaging (EPI) sequence to allow extended spatial coverage, which covers larger body regions without time penalty. Simultaneous acquisition of four slices was combined with segmented EPI for fast imaging with three gradient echo times in a preclinical perfusion study. Six female domestic pigs, German-landrace or hybrid-form, were scanned for 11 minutes respectively during administration of gadolinium-based contrast agent. Influences of reconstruction methods and training data were investigated. The separation into T1- and T2*-dependent signal contributions was achieved by fitting a standard analytical model to the acquired multi-echo data. The application of SMS yielded sufficient temporal resolution for the detection of the arterial input function in major vessels, while anatomical coverage allowed perfusion analysis of muscle tissue. The separation of the MR signal into T1- and T2*-dependent components allowed the correction of susceptibility related changes. We demonstrate a novel sequence for dynamic contrast-enhanced MRI that meets the requirements of temporal resolution (Δ*t* < 1.5 s) and image quality. The incorporation of SMS into multi-contrast, segmented EPI can overcome existing limitations of dynamic contrast enhancement and dynamic susceptibility contrast methods, when applied separately. The new approach allows both techniques to be combined in a single acquisition with a large spatial coverage.

## Introduction

Dynamic magnetic resonance imaging (MRI) has become an established imaging modality to assess a variety of physiological characteristics, such as tissue perfusion or metabolism in diseases [1]. Its potential to characterize tumor tissue with high image quality and reliability has made MRI the leading modality in modern radiology for this purpose [2]. Tumor vessels are highly disordered: they are characterized by dilatations, irregular diameters, excessive branching and arterial-venous shunts. Thus, tumor blood flow is chaotic and highly variable [3,4]. Dynamic MRI techniques allow assessment of tumor perfusion and vascularization. Various methods for dynamic MRI have been proposed and have coexisted for perfusion imaging over the last decades. The two most commonly used approaches for dynamic MRI with contrast agent (CA) enhancement are: dynamic contrast enhanced imaging (DCE), which relies on the T1-weighted signal, and the dynamic susceptibility contrast (DSC) method, which measures the T2- or T2*-weighted signal changes over time [5].

DCE and DSC (i.e. T1- and T2*-weighted methods) differ in the effect of CA on the MR signal, which is increased in DCE (due to reduced T1) and decreased in DSC (due to decreased T2*). There are differences between the methods regarding the underlying mechanisms which reflect different physiological information. T2*-change is a long-range phenomenon where sensitivity is dominated by the susceptibility gradient between regions of higher and lower CA concentrations (i.e. intravascular and extravascular extracellular space (EES) or during bolus phase [6]). By contrast, the T1-weighted contrast mechanism is more localized to tracer molecules and performs well in leakage quantification [6]. In particular, in low perfused tissue it is difficult to separate intravascular from extravascular signal contributions by one contrast alone (e.g. with DCE MRI [7]). The simultaneous acquisition of different contrast weightings allows removal of T2*-shortening effects from the T1-weighted DCE signal and vice versa [8–10]. Therefore, the separation of the measured MR signal into its T1- and T2*-dominated contributions reveals a more complete basis for physiological analysis [11]. This increases the reliability of calculated parameters, e.g. by using T1-dominated signal enhancement without T2* contribution to reduce the underestimate of blood volume in DSC [11].

The presented method of simultaneous multislice acquisition (SMS) using segmented echo-planar imaging (EPI) addresses the demand for the simultaneous measurement of multiple contrasts with a high temporal resolution. Insufficient temporal resolution often restricts advanced quantitative pharmacokinetic modelling in particular for dynamic contrast-enhanced MRI of large volumes [6,11,12]. During the early phase of the bolus arrival or for CA with high molecular weight the tracer remains intravascular and high susceptibility gradients dominate the formation of the MR signal [5,13]. Thus, T2*-weighted imaging allows the quantification of the arterial input function (AIF) if the temporal resolution is Δ*t* ≤ 1.5 s as suggested for accurate AIF sampling [6,12,14,15]. CA with low molecular weight diffuses into the tissue soon after injection and requires a two-compartment kinetic model to correctly describe the T2*-effects [9,16]. T2*-dependencies to the signal can be removed by extrapolation of the MR signal to TE = 0 ms [9]. The calculated signal S_0_ is primarily influenced by T1 and the localized, short range character of T1 relaxation gives insight into extravasation of the CA and permeability between vessels and EES [5,6,8]. Both contrasts can be used for pharmacokinetic modelling, thus providing additional information. The pharmacokinetic modelling itself is beyond the scope of this work.

In MRI sequence design there is a trade-off between high temporal resolution, which can typically be achieved by using a single-shot EPI readout, and short echo times. An echo time below 10 ms, which provides sufficient T1-weighting whilst maintaining a high temporal resolution, can be accomplished by segmentation of the EPI readout. Furthermore, the extension of segmented EPI to a multi-echo readout allows a flexible choice of crucial imaging parameters, such as temporal resolution, echo times and number of echoes. The optimal parameter values of these parameters might vary for different anatomies and expected dynamic range of T1 and T2*, allowing additional applications, such as bolus-tracking in a major vessel.

In the past decade, several challenges in simultaneous acquisition of multiple slices in 2D MRI have been overcome. In particular, since the blipped-CAIPI technique solved phase accumulation issues during EPI-readout [17] and the slice-GRAPPA (SG) algorithm was introduced [17], SMS has been widely used in brain imaging.

In this work, the acceleration of MRI by SMS is combined with the measurement of multiple gradient echoes in a flexible sequence design. This allows dynamic imaging of relatively large volumes outside the brain and the separation of mainly T1- and T2*-weighted MR signal contributions for advanced pharmacokinetic modelling. In the following sections, we provide details of the MR sequence design and different reconstruction approaches for SMS data, followed by an account of its application in-vivo.

## Materials and methods

### Sequence design

To image multiple echoes in multiple slices simultaneously, SMS was introduced to a multi-shot EPI sequence with segmentation along the phase-encoding direction [18] which was extended to several readouts [19]. This offers a flexible design in terms of spatial and temporal resolution with regard to desired image contrasts. A modulated multiband (MB) radiofrequency (RF)-pulse was chosen for SMS excitation. The positions of the individual slices contributing to the excited MB slice group are defined by a composite RF-pulse with individual slice-dependent components with offsets Δω and phases φ [20]:

$${RF}_{MB}=A(t)\;\cdot \sum_{n=0}^{N}\text{exp}\left(i\;\Delta \omega_{n}t+\varphi_{n}\right).$$ (1)

**Fig 1:**
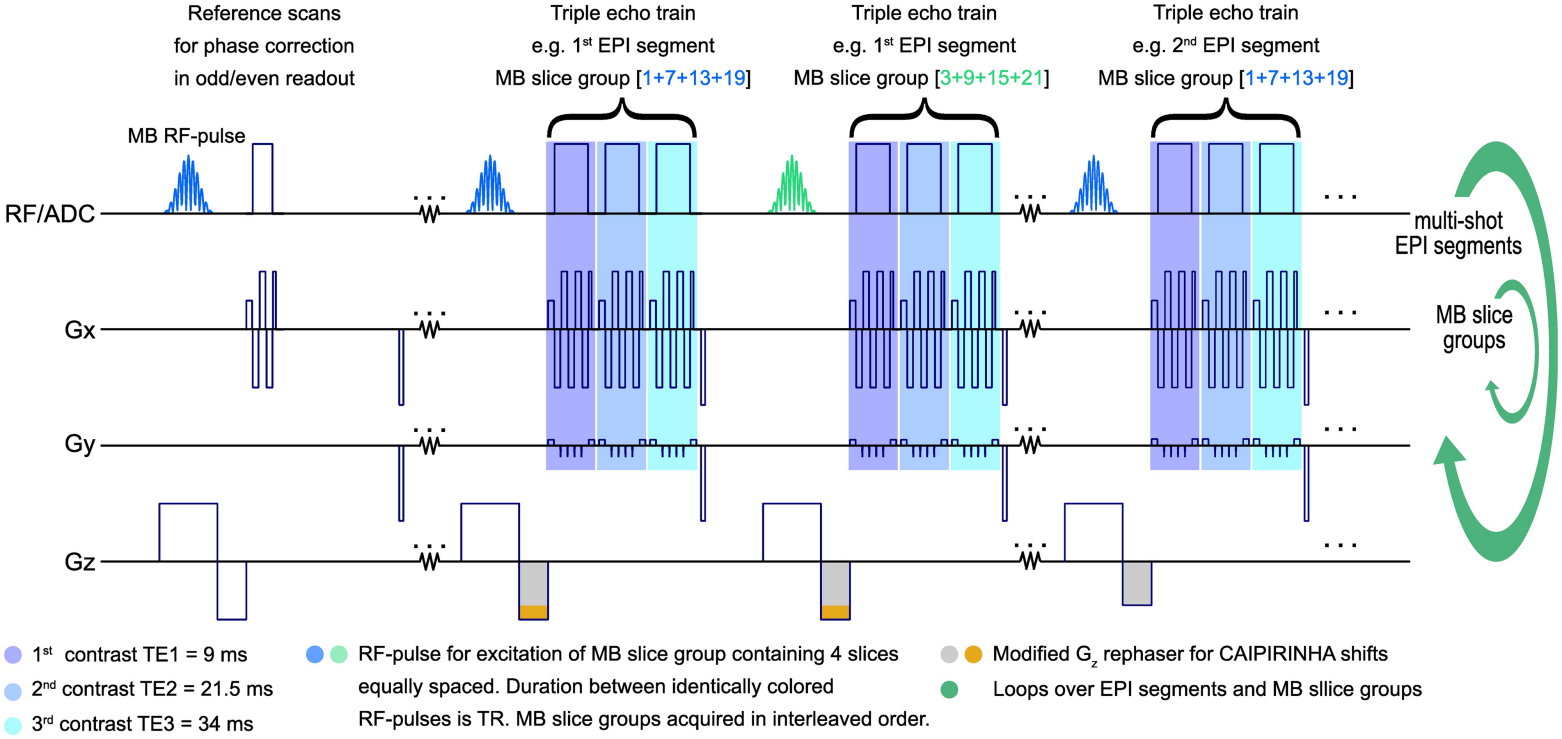
Sequence diagram for SMS acquisition using a segmented EPI with multiple contrasts. Looping is done over MB slice groups first, before looping over the EPI segments, to prevent from inter-slice contrast variation. An interleaved acquisition scheme was chosen to reduce cross talk between MB slice groups. CAIPIRINHA shifts: The orange area in G_z_ indicates the phase difference between EPI segments resulting in a phase modulation along PE depending on the z-position.

*A(t)* can be a standard complex waveform to define the desired slice profile. As such, all N simultaneously excited slices are expected to have identical flip angle, slice thickness Δ*z* and profile. CAIPIRINHA (controlled aliasing in parallel imaging results in higher acceleration) [21] was used to improve slice separation and to reduce SNR losses due to high geometry (g)-factors [17]. This was implemented by applying incomplete rephasing as a function of slice position and excited EPI segment, which leads to shifts of the overlapping SMS data along the phase-encoding (PE) direction. The modified slice-rephase gradients are highlighted in Fig 1. The phase modulation depends on the gradient moment difference defined by the current EPI segment i, counting from 0 to the number of segments *N*_*seg*_ *-1*, and the shift factor *FOV*_*shift*_, which describes by how much overlapping pixels are shifted along the PE direction. The modulation factor $s_{i}=i\;∙[i\;mod\;(\frac{1}{{FOV}_{shift}})]$ adjusts the additional phase depending on the acquired EPI segment and the desired shift.

As shown in Eq 2 the gradient of the slice-rephase gradient *G*_z,re_ differs from the slice-selection gradient *G*_z,se_ during the excitation of the i^th^ EPI segment which gives a locally defined phase difference:

$$\gamma \;\int_{t_{i}}^{T\;}G_{z,re}\;dt=\gamma \;\int_{t_{i}}^{T\;}G_{z,se}\;dt-\Delta \varphi_{z,CAIPI}$$ (2)

with the phase modulation function for the CAIPIRINHA shift

$$\Delta \varphi_{z,CAIPI}=\gamma {\cdot FOV}_{shift}\cdot z_{gap}\cdot s_{i},$$ (3)

where *z*_gap_ is the distance between the equally spaced slices within one MB slice group.

Autocalibration signals (ACS) similar to conventional in-plane GRAPPA [20] need to be acquired for the calculation of the weights in the SG reconstruction kernel. The dependence of the SG kernel on the underlying images has been reported [17]. Therefore, the SG kernel was calculated with additionally acquired ACS training data, where all parameters defining the image contrast, such as echo time (TE), flip angle (FA) and repetition time (TR), were identical for the SMS (ACS source) and the single-band (ACS target) training data. The resulting slice GRAPPA kernel was used for all SMS reconstructions of the following dynamic measurement.

**Fig 2:**
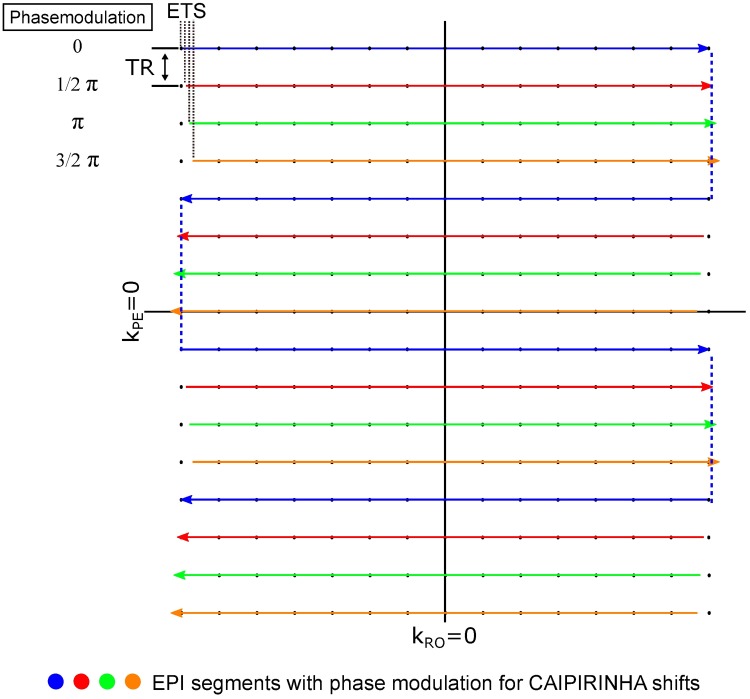
Segmentation of k-space into shorter echo trains allows a flexible timing scheme, short echo times and the acquisition of multiple contrasts even for signals with fast T2* decay. The duration between the EPI segments, i.e. acquisitions of adjacent k_y_ lines, equals the repetition time (TR). Slice group looping is done before the looping of the EPI segments.

At the beginning of measurement, additional phase correction data are measured. Three navigator lines (2 x positive, 1 x negative polarity of RO gradient) without PE are acquired during this reference scan to extract phase information and compensate for odd and even echo alternation. These navigator lines are excited by a MB pulse and used for MB slice group specific phase correction of the image data. The correction procedure is provided by the vendor’s image reconstruction system and consists of a linear phase correction succeeded by correction of the constant phase response [22]. An interleaved EPI acquisition scheme for PE-lines in k-space suffers from discontinuities along PE. The phase evolutions demonstrate a stair-step pattern, which might lead to severe image artifacts [23]. Echo time shifting (ETS in Fig 2) of 150 μs between adjacent EPI segments was used to reduce this artifact [24].

As indicated in Fig 2, the duration between successive excitations of the same volume, which allows longitudinal relaxation, is referred to as TR while the time, needed to acquire one complete set of data to reconstruct the individual slices, is defined as the temporal resolution *Δt*.

### Experimental setup

The performance of the MR sequence under preclinical conditions was evaluated by dynamic in-vivo imaging of adult pigs (German Landrace, approximately 20 weeks old, body weight of 52 kg to 64 kg, no food restriction). Six subjects were investigated, whereby four experiments could be run successfully without any experimental complications. The experimental procedure for dynamic CA-enhanced MRI with SMS and image reconstruction are described in the following sections. Details on animal handling and care, of the surgical technique as well as results of previous MRI experiments performed with this animal model are described elsewhere [25].

All experiments were performed in accordance with the German animal protection law and ethical approval was provided by the Landesamt fuer Natur, Umwelt und Verbraucherschutz Nordrhein-Westfalen, Germany (No: 84–02.04.2012.A208). The animals’ heart rates and oxygen saturations were monitored with a MRI-compatible device (Verdis, Medrad, Germany) during surgery and MRI examination. The pig was euthanized under higher level of anesthesia by injection of T-61 (0.3 ml/kg) after the experiment [25].

### Imaging parameters and protocol

Measurements were performed at 1.5 T using a commercial MRI system (Magnetom Aera, Siemens Healthcare, Erlangen, Germany) in a clinical environment. The subject was set in a supine position allowing placement of two phased-array coils (18+12 channels) around the hip and leg region. Additionally, four 4-channel spine coils were used, giving a total of 46 receiver channels which was the hardware limit of the MRI system used. High-resolution T1-weighted turbo spin-echo images (TSE; TR = 559 ms, TE = 9.7 ms, resolution: 0.8 x 0.8 x 5 mm^3^) in coronal, transversal and sagittal orientation were acquired to ensure correct positioning of the FOV. ACS data were collected before bolus injection and with consideration of their contrast. To ensure steady-state conditions, only the last one of the three directly repeated measurements was used as ACS data for calculation of the SMS reconstruction kernel.

To cover the hip and leg on one side of the animal in a sagittal orientation a FOV of 400 x 200 mm^2^ was selected. With respect to the desired temporal resolution of Δ*t* ≤ 1.5 s [6,12,14,15], the sequence described above allowed the acquisition of 128 x 68 pixels in-plane at a spatial resolution of 3.1 x 3.1 mm^2^ in 24 slices of 5 mm thickness (40% gap) due to a four-fold accelerated imaging speed (*R* = 4). Acceleration was achieved by SMS with MB = 4 only, without any in-plane undersampling. The repetition time could be reduced to TR = 261 ms with acquisition of a triple echo train (TE1 = 9 ms, TE2 = 21.5 ms, TE3 = 34 ms). Four EPI segments were acquired, each with an echo train length of ETL = 17. A CAIPIRINHA shift of FOV/4 between slices in one MB slice group was applied. The sequence design was optimized to reduce inter-slice contrast variation (Fig 1) and to keep TR sufficiently long to recover more magnetization before the next excitation (Fig 2). Cross-talk of adjacent MB slice groups was counteracted by an interleaved sampling scheme (Fig 1). Other sequence parameters were: *FA* = 90°, echo-spacing 0.61 ms, and bandwidth 2056 Hz/Px.

The CA (Dotarem, Guerbet, Villepinte, France) bolus was injected into the jugular vein through a central venous catheter after the 5^th^ dynamic measurement and automatically controlled by a power injector (Medrad Spectris Solaris EP, Bayer AG, Leverkusen, Germany) using a dosage of 0.1 mmol/kg at a flow rate of 5 ml/s followed by a 20 ml (5 ml/s) flush of saline solution (0.9%). The total scan time for 500 acquisitions at a temporal resolution of Δ*t* = 1.305 s was 10:52 minutes.

### Postprocessing

Image reconstruction was conducted offline, because online reconstruction for SMS was not available for the system’s software (syngio MR D13A, Siemens Healthcare, Erlangen, Germany) at the time when experiments were run. Moreover, offline postprocessing ensures full control of the reconstruction and comparison of different reconstruction approaches. First, the raw data were preprocessed in the vendor’s image calculation environment (ICE, Siemens Healthcare, Erlangen, Germany). Preprocessing included Nyquist-ghost correction of the collapsed SMS data. An export tool saved the preprocessed data for further image computing.

Data reorganization, preparation of ACS, calculation of the SMS reconstruction kernel and unfolding of the measured SMS datasets were performed in MATLAB (MathWorks, Natick, MA). Implementations of two algorithms to separate overlapping pixel contributions in the MB slice group were compared: SG [17] and split slice-GRAPPA (SSG), also known as leak-block slice-GRAPPA [26].

After the SMS reconstruction, the images of individual receiver channels were merged by sum-of-squares combination. The unfolded images were passed to MeVisLab (MeVis Medical Solutions AG, Bremen, Germany) [27] for further image processing. The three different echo times of each image of the time series were fitted to a mono-exponential T2*-decay

$$S(t)=S_{0}\cdot e^{-\frac{TE}{T_{2}^{*}}}$$ (4)

to provide the extrapolated dynamic T1-weighted MR signal *S*_0_ at TE = 0 ms and the corresponding T2*-dynamics [8]. The implemented fitting routine uses a Levenberg-Marquardt algorithm provided by the MPFIT-library [28]. Initial values were automatically estimated by linear regression.

### Image reconstruction and quality assessment

As in [17,20,26], single-band (SB) data were acquired with the same in-plane imaging parameters as the SMS measurement and serve for training of the reconstruction kernel. Because of the continuous and smooth variation of the coil sensitivities across the FOV, low resolution ACS data can be used to train the reconstruction kernels [20]. ACS data with higher resolution did not improve the reconstruction result any further, but demanded more computing power. Here, the full-resolution SB data were downsampled in a postprocessing step (64 x 64 in-plane) and used for ACS. Two different methods for the formation of the ACS for SG were tested. In the first method ACS target data were derived from an SB acquisition (TR = 1500 ms) and shifted according to the CAIPIRINHA scheme. Measured data of a SMS acquisition with *MB* = 4 (TR = 1500 ms) were used as ACS source data. For the second method, ACS source and target data were delivered by the same SB acquisition (TR = 1500 ms) and, in contrast to the first method, ACS source data were not measured directly as SMS, but created by collapsing specific slices into a synthesized MB slice group. Furthermore, we compared the performance of the SG to the SSG algorithm. The ACS for SSG was chosen as in the second method only as proposed in [26], because the SSG algorithm can not use unseparated, measured ACS source data for kernel calculation. The reconstructed SMS images *I*_SMS_ for the different reconstruction approaches and the reference SB images *I*_SB_ were compared. Normalized subtraction maps revealed local reconstruction errors

$$E_{\text{diff}}=M_{b,H}\;|\frac{I_{\text{SMS}}-I_{\text{SB}}}{I_{\text{SB}}}|$$ (5)

and after removal of background noise by binary-masking, the mean error across the volume quantifies the exactness of reconstruction. A Huang-threshold was applied to the image-volume to derive a binary mask *M*_b,H_ [29]. Localized differences between the reconstructed SMS and the reference SB data might have severe impact on quantitative analysis and should be minimized while homogeneous differences are acceptable and constant offsets can be easily removed by scaling.

Furthermore, the g-factor [20,30], as a quantitative measure to describe the unfolding process and related noise amplification, was computed for the different approaches and its mean and standard deviation across the object were calculated. Both metrics, the normalized subtraction error and the g-factor, are displayed exemplarily as maps for one MB slice group together with histograms and the corresponding mean values for all 24 slices.

It should be noted, that the performance of SMS reconstructions is directly related to the positioning of the receiver coils which is not fully reproducible if flexible phased-array coils are used as in the presented in-vivo experiments.

As reported in previous publications [17], the reconstruction of SMS data with SG or SSG may have some dependency on the image contrast of the training data. This is of special interest in case of CA enhanced measurements where SMS image data vary contrast throughout the dynamic scan. An analysis of the reported contrast dependency of SG / SSG was conducted in additional ex-vivo experiments on a phantom. Specifically, possible dependencies on the image parameters of interest, namely base signal S_0_ and T2* (Eq 4), were closely investigated. One reconstruction where ACS and SMS data have identical image contrast, which is considered as ground truth, was compared to another reconstruction where the image contrast of ACS and SMS data show different image contrast, see S1 Fig. The normalized difference in the image signal ΔS, defined as

$$\Delta S=\frac{\left(S_{A}-S_{B}\right)}{S_{A}},$$ (6)

was considered with respect to S_0_ and T2*, where the indices A, B indicate differences in the ACS data used for reconstruction. The observed discrepancies between the reconstructions, with (A) and without (B) identical image contrast in ACS and SMS data, were evaluated by a linear model (b_0_, b_1_).

The influence of image contrast and SNR (1, 5 and 10 averages) from the type of ACS data used were compared. Single-slice measurements of a structured phantom (T1_phantom_ = 289 ms) containing contrast-agent doped sub-volumes (T1_subVol_ = 211 / 434 / 556 / 953 / 1257 ms) were used for evaluation. SMS data with CAIPIRINHA shifts were synthesized (see above) to analyze effects of the SG and SSG algorithms independently from the SMS sequence or other fluctuations. The signal levels for the sequence parameters to vary (FA) were simulated beforehand to estimate resulting contrast (S1 Fig). Data with FA = 5°, TR = 30 ms were reconstructed with SG/SSG using kernels calculated from either ACS with the same imaging parameters (FA = 5°, TR = 30 ms) as suggested by [17] or from ACS with different image contrasts (FA = 90°, TR = 30 ms). In addition, a comparison between individual kernels for each TE (ACS123) and a single reconstruction kernel (ACS111) derived only from the first echo, TE1, with highest SNR was performed.

## Results

The focus of this work is on the general method and, therefore, only the results of a single experiment are presented in detail. The findings are consistent with data derived from four similar experiments. Overviews of the results of the other three experiments are given in the supporting information.

### Signal separation and dynamic imaging

**Fig 3:**
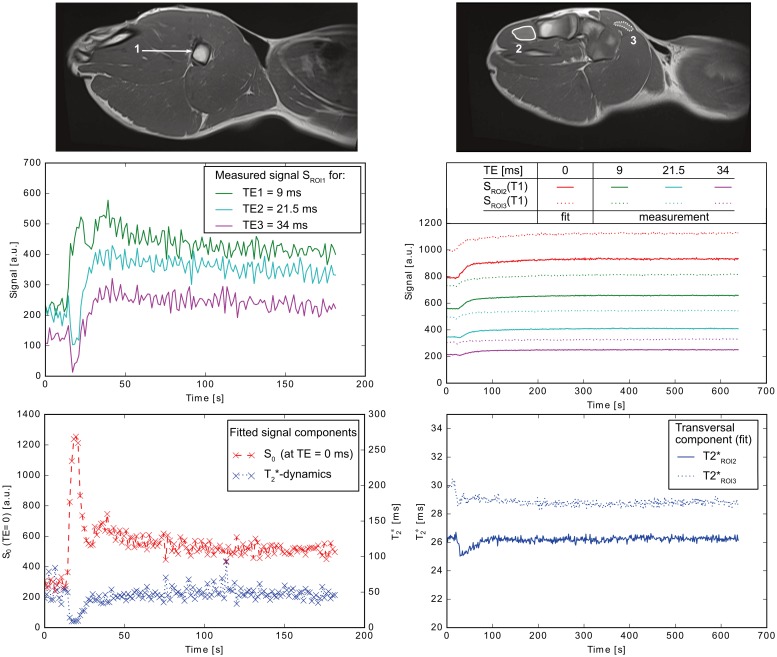
ROI1 represents a voxel dominated by intravascular signal. The intravascular signal describes typical AIF curve characteristics (left column, slice 15). In addition, representative regions in muscle tissue were selected for dynamic signal curves in tissue (right column, slice 19: ROI2 (solid), ROI3 (dotted)). High resolution anatomical images (TSE, sagittal) were used for selection and positioning of the ROIs. The diagrams in the left column show the signal dynamics in a major vessel for all three acquired contrasts (middle row) as well as for the separated signal components (bottom row). In the right column, the signals in tissue ROIs are displayed. For ROI2 (solid) and ROI3 (dotted) the three acquired contrasts (TE1, TE2, TE3) and the extrapolated signal for TE = 0 ms are depicted (middle row). The changes in T2* are shown in the bottom row. The sensitivity of T2* to inflow effects of CA leads to a decreased signal, which recovers to a constant level when CA is distributed in the ROI.

500 repeated measurements from the dynamic protocol under CA administration were reconstructed with a fixed, precalculated SG kernel. The SG kernel was based on separate ACS source (*MB* = 4) and target (SB) data with identical image contrast parameters (method 1, ACS111) which were acquired before CA administration. Fig 3 (top row) shows high-resolution TSE images, acquired prior to the dynamic scans with identical slice positioning to ease the definition of regions of interest (ROIs). The left column shows the signal from a single voxel (ROI1). ROI1 is located in a major vessel where a direct response to the inflowing CA can be easily identified. The signal time-course for all three contrasts in the early phase (200 s) is shown in the middle row (left column). During the CA-bolus, the T2*-effects dominate, even in the early contrasts, which have stronger T1-weighting. By the signal separation procedure based on Eq 4, T2*-contributions are removed and an essentially T1-weighted signal S_0_ (TE = 0 ms) can be recovered. The subfigure in the bottom row of Fig 3 (left column) shows the increasing S_0_-signal, despite the reduction of the exponential decay time T2* in presents of a CA. Two additional ROIs were selected from homogeneous regions in muscle tissue (Fig 3, right column). Their signal-time courses as well as the resulting S_0_ dynamics are shown in the middle row (right column). The measured signals for TE1, TE2, TE3 are consequently lower than S_0_ at TE = 0 ms. The corresponding T2*-values in both ROIs for all 500 measurements are shown in the bottom row (right column). The signal decreases compared to S_0_ because of susceptibility effects during CA arrival. This reveals the measurements sensitivity to CA-effects. After some time (> 100 s), T2* continues at a constant level when the CA is distributed equally in intra- and extravascular space.

### Reconstruction performance and image quality

**Fig 4:**
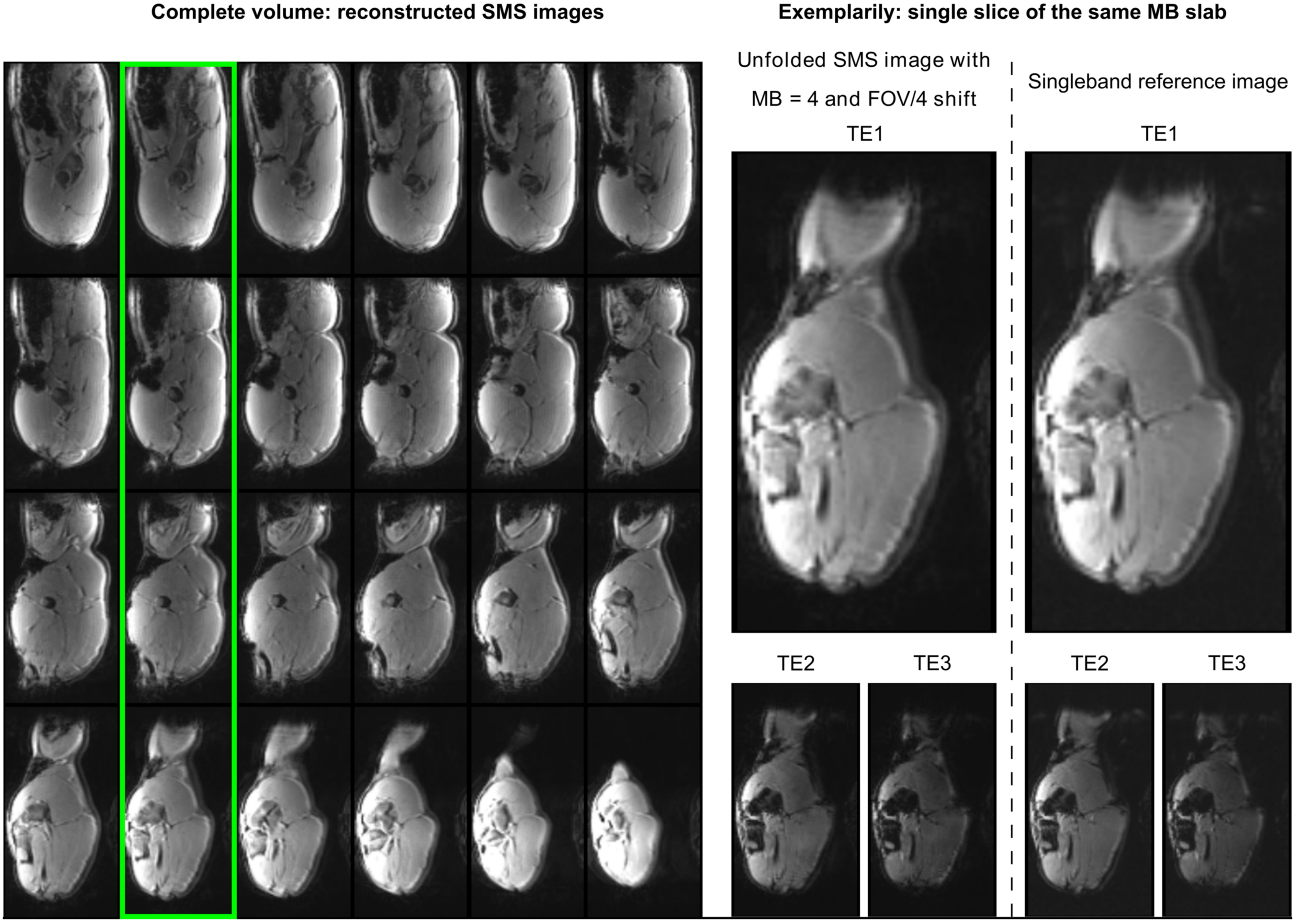
SMS image reconstruction of the complete volume (24 slices, MB = 4, FOV/4 shift) performed with SG [17]. For kernel training measured SMS and SB data with identical imaging parameters were used. Four representative slices from one MB slice group are selected for further illustration (green box). A single slice with a TE1 = 9 ms of the reconstructed SMS image (center) and the reconstruction of the SB reference data (right) are shown in zoomed view. The results for the other echoes, TE2 and TE3, are depicted below the TE1 images, accordingly.

Fig 4 shows the 4x accelerated SMS acquisitions after unfolding and reconstruction. All 24 slices of the volume are displayed for completeness. In later figures only representative slices from one MB slice group are selected for clarity.

The quality of the SMS images is comparable to the SB images which were acquired with the same parameters: TE1 = 9 ms and TR = 1500 ms (Fig 4, right top row). A similar reconstruction performance could be achieved for the later echo times as depicted in Fig 4 (right bottom row). No image normalization was applied, so prominent shading effects, reflecting the positioning of the phased array receiver coils, appear in all cases.

**Fig 5:**
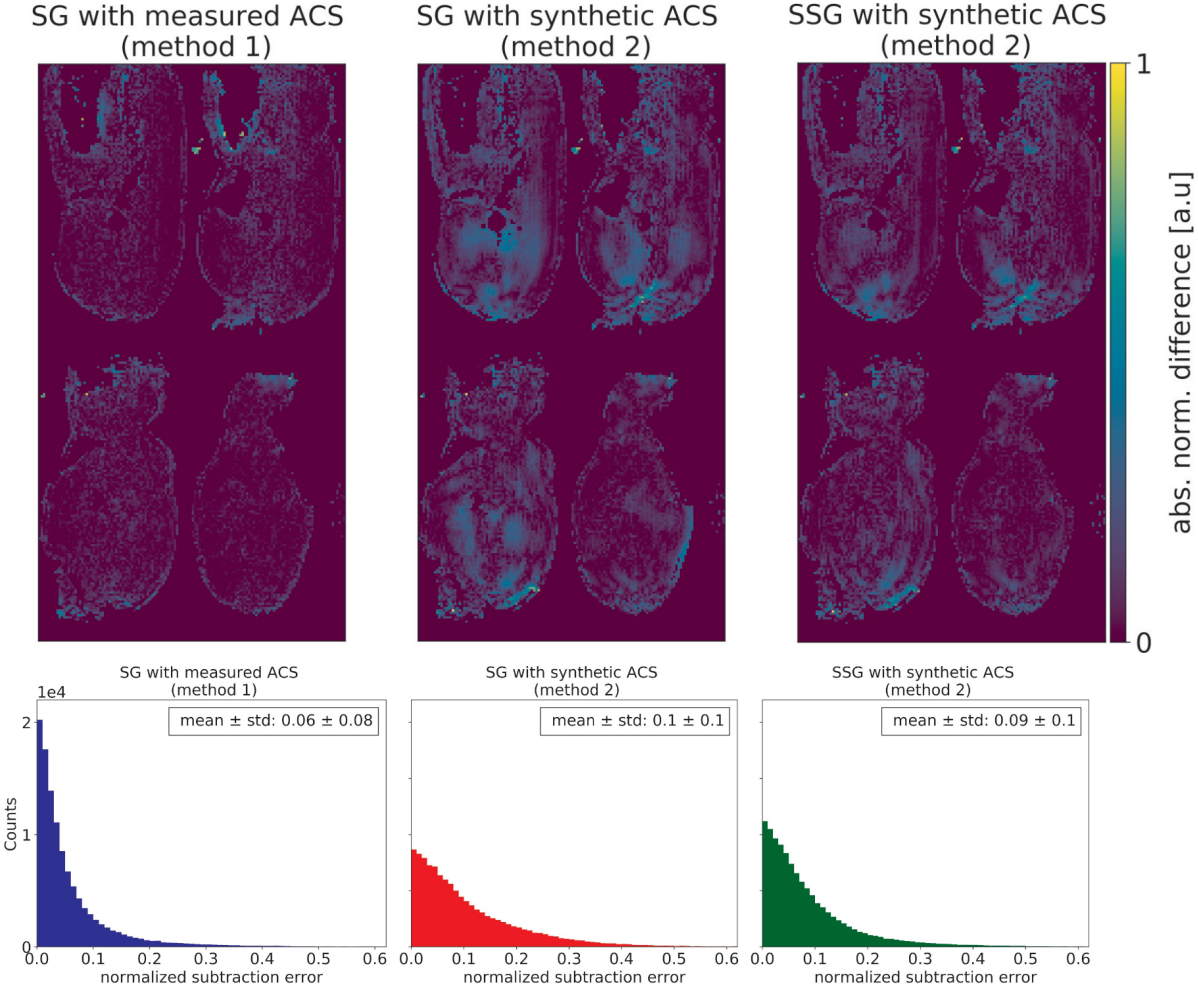
The normalized subtraction maps of the different reconstruction methods are shown. From left to right: Method 1, where the SG algorithm with measured data for ACS source (MB = 4) and ACS target (SB) was used for reconstruction. Method 2 (center), where the reconstruction is performed with synthesized ACS source data and, for comparison, a reconstruction by the SSG algorithm (right) [26]. The most homogenous reconstruction result can be achieved by method 1 (left), while method 2, especially for the SG algorithm, shows strong localized differences. The mean errors across the volume, after masking to remove noise and the corresponding histogram, are below each map, respectively.

In Fig 5, the two alternative reconstructions methods for SG and the reconstruction with SSG (method 2 only) are compared. Method 1 (left), where ACS source data were measured with SMS (*MB* = 4) and the same parameters as for the SB acquisition, shows the most accurate reconstruction. The normalized subtraction map shows a homogenous reconstruction of the signal throughout the recovered slices. For the SMS reconstruction with SG (method 1) the mean error across the volume is ${\bar{E}}_{diff,SG,1}=0.06$. In comparison, the reconstruction with method 2 for SG (center) suffers from localized intra- and inter-slice differences. A mean error of ${\bar{E}}_{diff,SG,2}=0.1$ was determined in this case. The use of the SSG algorithm (right) addresses slice leakage reconstruction errors and reduces inhomogeneity if compared to SG (method 2), but remaining local errors with a mean of ${\bar{E}}_{diff,\text{SSG},2}=0.09$ can be identified.

**Fig 6:**
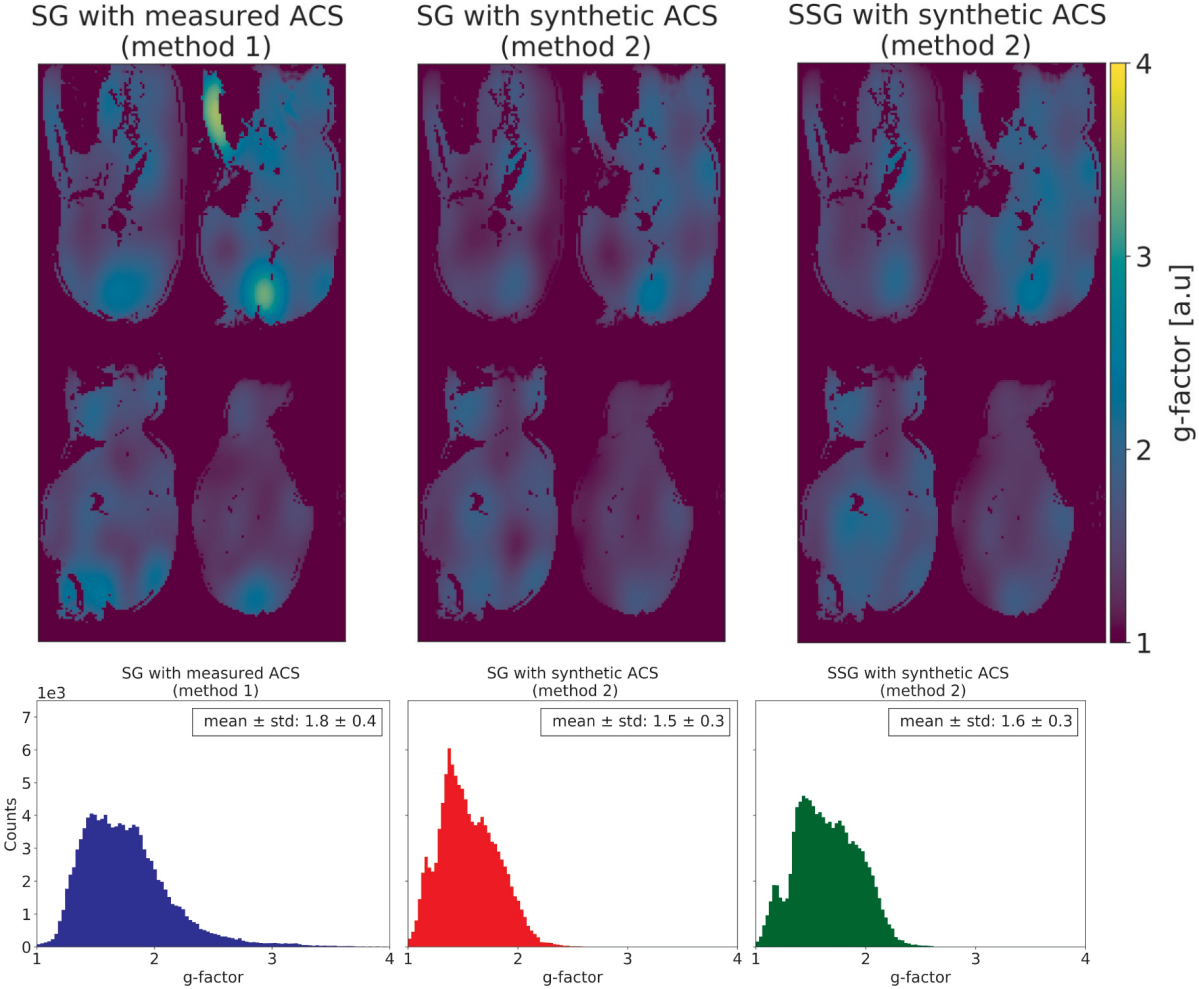
The g-factors of the different reconstruction approaches were calculated as suggested in [30] and displayed for completeness. The mean g-factor across the object region are in the same range for the compared approaches, with the chosen reconstruction strategy (left) indicates a slightly higher g-factor than the two others. The corresponding histograms as well as the mean g-factor for all slices are displayed below each map.

For completeness, reconstruction-related noise enhancement, which is characterized by the g-factor, is depicted in Fig 6. G-factors larger than 1 indicate that SNR was (locally) reduced by the reconstruction procedure. The mean g-factors for the compared reconstruction methods are in a close range, with 1.8 ± 0.4 for method 1 (SG) and 1.5 ± 0.3 for method 2 (SG), respectively. While the g-factor for the SSG reconstruction (method 2) is 1.6 ± 0.3.

**Fig 7:**
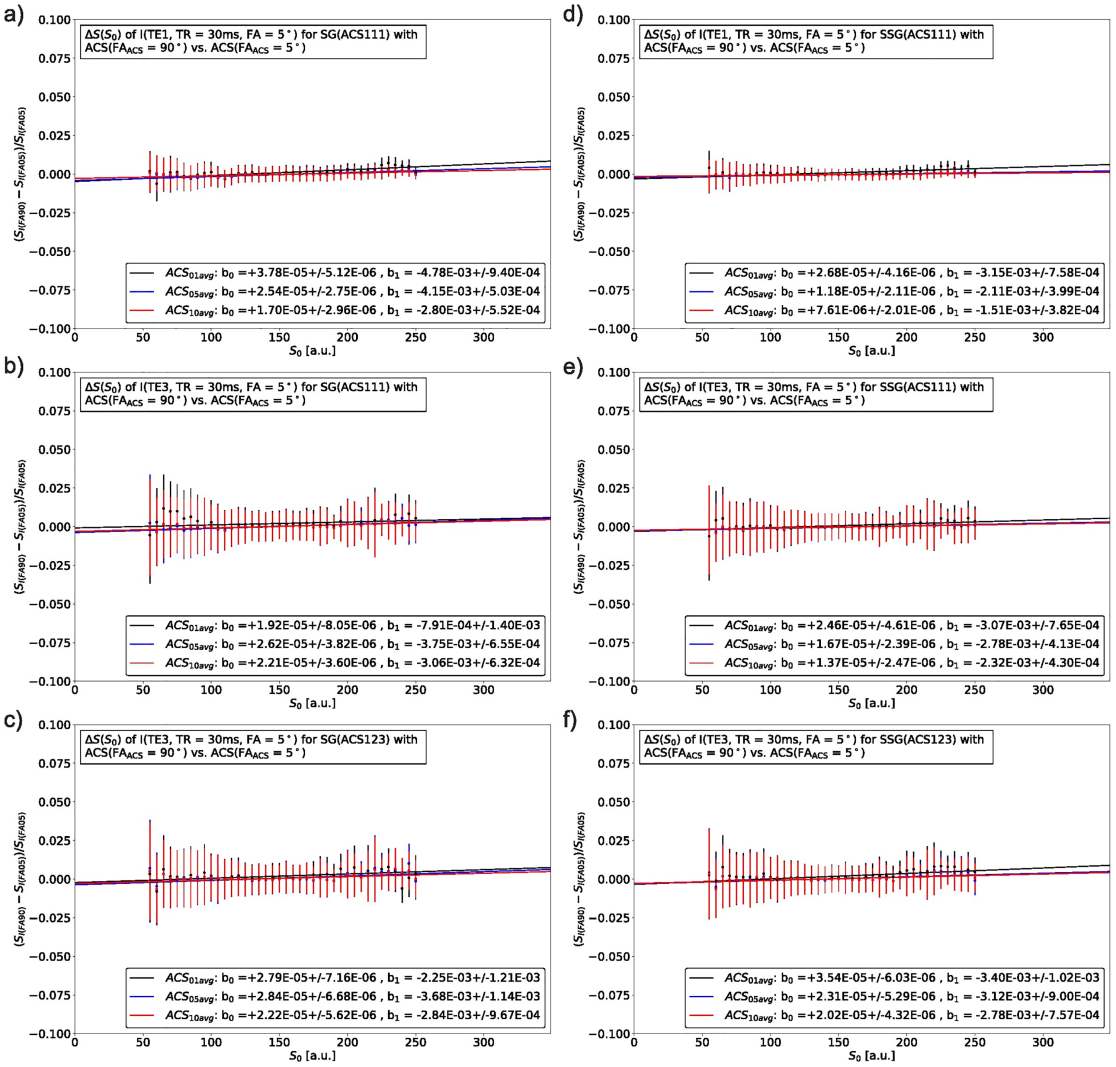
Synthetic SMS images (FA = 5°, TR = 30 ms) were disentangled with reconstruction kernels calculated from either ACS with identical (FA = 5°, TR = 30 ms) or different image contrast (FA = 90°, TR = 30 ms). The resulting normalized signal differences for SG (left) and SSG (right) with respect to the base signal S_0_ are shown for three levels of SNR in the ACS (1, 5 and 8 averages). Subfigure (a) and (b) depict the first (TE1) and third echo (TE3) when reconstructed with a single, constant kernel (ACS111). In (c) each echo is reconstructed with its corresponding echo in the ACS (ACS123). The subfigures (d), (e) and (f) illustrate these dependencies for SSG. S_0_-values along the x-axis were binned to a width of 5. On the y-axis are the normalized differences between both reconstructions as formulated in Eq 6, the vertical bars represent the standard deviation of the mean value after binning.

**Fig 8:**
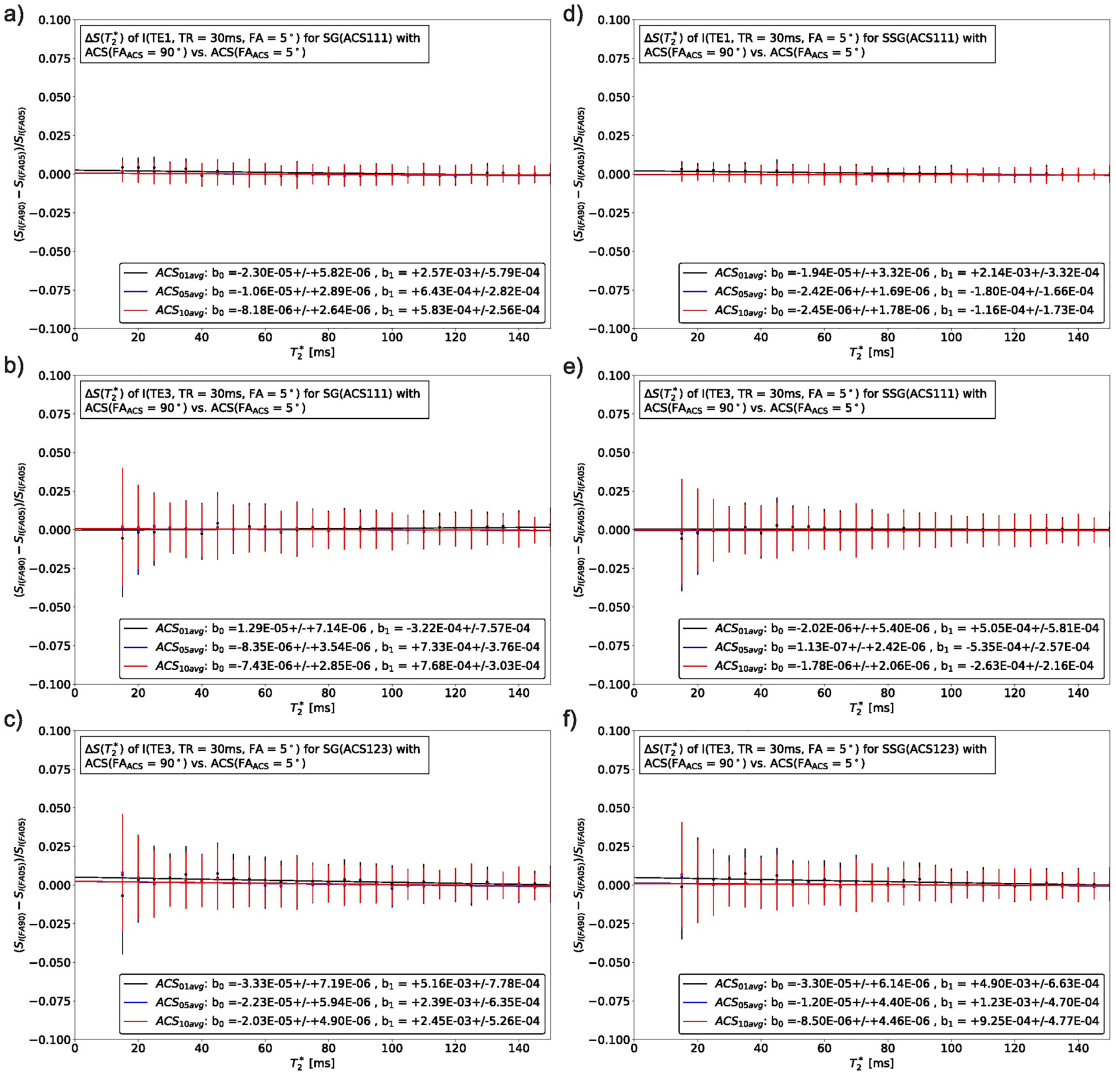
The T2*-dependency of the image signal for unwrapped, synthetic SMS images (FA = 5°, TR = 50 ms) is shown for SG (left) and SSG (right). As in Fig 7 the reconstruction results of different ACS are compared by subtraction. While (a), (b), (d) and (e) represent reconstructions where ACS is from the first echo only (ACS111), the subfigures (c) and (f) show SG and SSG reconstructions for TE3 when each echo receives its own reconstruction kernel (ACS123). The bin-width is 5 ms. On the y-axis are the normalized differences between both reconstructions as formulated in Eq 6, the vertical bars represent the standard deviation of the mean value after binning.

**Fig 9:**
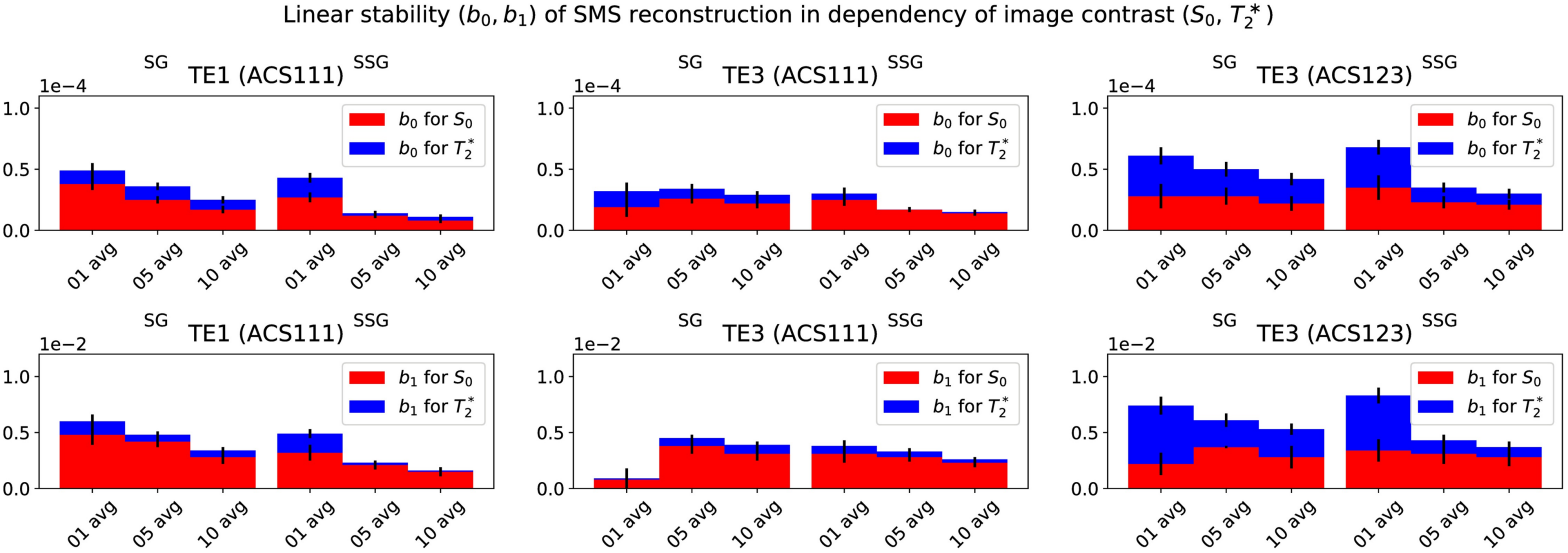
As shown in Figs 7 and 8, the robustness of the reconstruction with respect to S_0_ (red) and T_2_* (blue) was quantified by a linear model (b_0_, b_1_). Besides different combinations of the ACS training data, three different SNR-levels of the ACS data are shown (1, 5 and 10 averages) for SG (left) and SSG (right). The slopes, b_0_, for both contrasts, S_0_ and T_2_*, are visualized in the top row for the compared training configurations and TEs. The bottom row displays the offsets, b_1_, of the linear model.

To evaluate potential dependencies of the SMS reconstruction on the underlying image contrast, ACS with different image contrast were compared in their performance and influence on the reconstruction result. Figs 7 and 8 display the S_0_ and T2* dependency for different combinations of ACS for SG and SSG reconstructions. For each combination different color-coded SNR levels were also plotted: 1 avg. (black), 5 avg. (blue), and 10 avg. (red). The robustness of the tested reconstruction strategies was quantified by a linear model (b_0_, b_1_), where the resulting fitted parameters are shown in the respective figures. A detailed overview of the results is given in Fig 9 and S1 Table.

## Discussion

The presented SMS multi-contrast segmented EPI sequence allows fast dynamic imaging of a large volume of interest. Its performance for dynamic MRI was tested in a preclinical perfusion study under CA administration. A multi-shot segmented EPI sequence yields sufficient signal at late echo times for T2*-contrast, as well as providing short-echo time data for T1-weighted contrast. The segmentation of the echo train offers an additional parameter, which allows flexible adjustment of the acquisition scheme with respect to both, the desired echo times and the temporal and spatial resolution for a given volume of interest. This work has shown that the combination of SMS and multi-echo, segmented EPI permits comprehensive dynamic imaging studies with scan times that would be acceptable in clinical practice. Here, a volume of 400 x 200 mm^2^ was covered within the upper limit for the temporal resolution of Δ*t* ≤ 1.5 s for accurate AIF detection. The SMS protocol used in this study is consistent with other perfusion protocols for DCE, DSC [16] or combined T1/T2* imaging [10,31], but offers a higher flexibility than, for example, single-shot EPI, which is often limited to DSC in the brain [31]. The reconstructed SMS images do not suffer from any obvious residual inter-slice artifacts of the collapsed slices (Fig 5, left column), which confirms the correct unfolding of the accelerated SMS data.

Nyquist-ghosts occur in EPI sequences because of a temporal misalignment between alternating readout gradients and signal acquisition. If not corrected appropriately, this results in a N_FOV,PE_/2-ghost for single-shot EPI for a FOV of N_FOV,PE_ pixels along PE. In case of segmented EPI, the signal intensity of the ghost-artifact is shared between the N_seg_ segments resulting in N_seg_ ghosts distributed along N_FOV,PE_ [32]. The SMS reconstructions in this work do not suffer from obvious Nyquist-ghosting. However, if Nyquist-ghosts appear more pronounced, the slice separation procedure may precede the phase correction as suggested in [33].

Segmented sampling strategies have been restrained because of their severe vulnerability to motion, which, in particular, limits their application for abdominal or cardiac imaging by introduced structured artefacts. As structured artifacts due to motion are a drawback for all segmentation strategies, the achieved overall acceleration by SMS encounters motion related errors to a certain level. Nevertheless, the intrinsic sensitivity to any motion between the segments of the same frame of EPI data will remain a clear disadvantage. If motion can be controlled, e.g. data are acquired under breath hold, the field of applications may be extended and further acceleration as described later on may help to reduce present restrictions in the application of segmented EPI.

Standard parallel imaging (PI) strategies in (single-shot) EPI apply in-plane undersampling either merely for acceleration or also to reduce distortion and echo-time. In comparison to PI where an acceleration factor of R = 4 is selected, SMS measurements with R = 4 achieve a higher overall acceleration. The in-plane undersampling in PI only reduces the duration of the readout, whereas SMS shortens the complete TR period including the excitation and the readout. Moreover, with similar temporal and spatial performance, the introduction of SMS to multi-contrast perfusion MRI does not suffer from an $\sqrt{R}$-fold SNR penalty like with PI [20].

It should be emphasized, that PI can be incorporated into this type of sequence if further acceleration is desired, e.g. to reduce sensitivity to motion. PI accelerated acquisition of multi-contrast dynamic MRI has shown promising results in brain perfusion mapping [10]. Although, PI is an established technique, acceleration is limited due to inherent reduction in SNR by $\sqrt{R}$, in addition to reconstruction related SNR penalty (g-factor) [34]. The shortened read-out train and, therefore, a shorter TE are beneficial for some applications especially in single-shot EPI, but are less important if the readout is segmented as in the presented work. While the setup includes 46 receiver coils which would allow combined SMS and PI theoretically, this was not considered in the experiments. First, because the achieved temporal resolution met the prior defined requirements and second, because of potentially poor coil sensitivity profiles for the subjects’ anatomical conditions due to difficult placement of the coils and resulting spatially inhomogene-ous signal levels. Poor coil sensitivity profiles would affect SMS and PI in a similar manner, but the SG/SSG reconstruction in SMS exploits sensitivity variations along two directions, PE and slice, which typically reduces the g-factor penalty compared to parallel imaging reconstructions in 2D MRI where only coil sensitivity variations along PE direction are used [35].

The relatively high g-factors for the evaluated reconstruction approaches (Fig 6) lead to localized SNR penalties, which can be seen in the subtraction images for reconstructions where ACS data were synthesized (Fig 5, method 2). These prominent localized differences do not occur if the ACS source data used for training in SG are the measured SMS data. Therefore, the higher g-factor in method 1 than in method 2 was accepted. Method 1 provides a robust reconstruction with less localized inhomogeneities for the given experimental setups and no obvious inter-slice leakage was observed by visual inspection of the final images, which would have required slice leakage blocking. The dynamic dataset was reconstructed with a SG kernel calculated accordingly (method 1). The pixel-wise difference after subtraction (Fig 5) and the g-factor (Fig 6) represent two different metrics for the quantification of the reconstruction performance in SMS. Reasons for the disagreement in both metrics, i.e. lower g-factor vs. higher subtraction difference for method 2 (Figs 5 and 6), might arise from the algorithm for the g-factor calculation itself. It derives the g-factor from the variances of the accelerated and reference images which are gained from identical data in method 2 [30].

These results suggest that image quality is improved by acquiring additional low resolution training data: SB data for ACS target and data from a SMS acquisition serving as ACS source. In particular, for perfusion imaging or other protocols with repeated measurements, this extra time penalty might be acceptable. Additional experiments to analyze and verify this approach as well as an account on underlying reasons in real measurements are given in S10 Appendix. Note, that special care must be taken to avoid object motion between ACS scans. Motion and its artifacts are an issue which, in general, hamper dynamic MRI. Imaging methods where the reconstruction relies on reference data are inherently affected by motion. The repeated acquisition of reference data, for example, during the extravasation phase some time after the bolus, when temporal resolution is less critical, could help to ensure a correct match between reference and SMS data.

A CAIPIRINHA-like shifting by gradient modulated phases along PE reduces the g-factor penalty. Here, the modulation gradients were integrated into the rewinder of the slice-selection gradients. This novel method to introduce phase modulation can become an alternative to the z-blips commonly used in single-shot EPI, if the k-space is segmented along PE A drawback of this strategy is a limitation in *FOV*_*shift*_-factors selectable with respect to a given number of EPI segments. On the other hand, omitting extra gradients for shifting reduces gradient switching and avoids additional gradient blips, which can have disadvantages, such as eddy currents or increased echo spacing, as well as some signal loss due to intra-slice dephasing.

Influences of the SMS reconstruction process on the image contrast [17] have been addressed by the choice of ACS data with identical contrast and sufficiently high SNR (Figs 7 and 8). The evaluation of different strategies suggests, that the reconstruction performance for either SG or SSG depend more on a sufficiently high SNR than on image contrast, see Fig 9. Averaged ACS (5 or 10 averages) with higher SNR tend to reconstruct SMS data independently of the image contrast (TE1, TE3, S_0_, T2*). SSG is slightly more robust than SG, but with sufficient SNR the methods are comparable. It should be noted, that the resulting parameters of the linear model (b_0_, b_1_) which quantify this dependency are all relatively small and close to zero, indicating that there is no dependency on S_0_ or T2*. Hence, by correct choice of ACS data, a scaling of the image signal during SMS reconstruction, which appears when source and target ACS have different contrasts, can be avoided and the recovered SMS images exhibit the correct contrast according to their imaging parameters.

No additional inter-slice leakage or ghosting was observed during or after CA administration, which locally or globally changed the image contrast. This confirms that the reconstruction method performed equally well throughout the dynamic protocol, in line with the results reported previously [36].

An interleaved acquisition scheme for the MB slice groups was used to reduce cross-talk between adjacent slice groups and saturation effects in the magnetization. This ensures identical contrast characteristics across the volume.

The two major aspects motivating this work were analyzed. Firstly, the acquisition of multiple contrasts enables separation of the MR signal into multiple components by fitting a mono-exponential decay to the contrasts. The resulting S_0_-signal, which depends on mainly T1, and a T2*-dependent signal reflect different pharmacokinetic properties. The presented work qualitatively shows that signal separation can be successfully performed and the expected dynamics of S_0_ and T2* can be extracted. These relaxation times provide additional information for perfusion and extravasation modelling (e.g. a vessel-size index describing geometry might be calculated). Therefore, baseline measurements can be used for the quantification of the relaxation-time change rates and offer data for advanced pharmacokinetic modeling in future work. Secondly, an accelerated MR sequence for dynamic contrast-enhanced imaging at a temporal resolution of Δ*t* < 1.5 s with sufficient spatial resolution has been investigated. It allows sampling of the AIF in major vessels with a temporal resolution of Δ*t* = 1.305 s per image. The FOV available covered the pig’s leg and hip, including the abdominal aorta. The segmentation of the EPI readout allows the acquisition of the desired (short and long) echo times. By the acquisition of multiple contrasts, separated signals at TE = 0 ms can be recovered from a mono-exponential model to sample the AIF despite the fast signal decay during CA-bolus passage with its dominant T2*-effect.

AIF detection in large major vessels allows the minimization of partial-volume effects in the case of voxels which are completely intravascular. The signal drop during the bolus phase dominated in all three acquired contrasts and, thus, reliable separation of the signal contributions was difficult in the abdominal aorta with the chosen FA of 90° and the given CA concentration. This suggests the use of a lower FA for similar settings in the future. By the acquisition of multiple-contrast, the resulting concomitant T1 changes during the bolus phase can be avoided [37]. For other major vessels in the arterial tree the bolus dispersion allows detection of the AIF and signal separation as shown in Fig 3 (left).

Furthermore, the severe B1 errors introduced by the suboptimal choice of FA can be reduced by a reduction of the applied FA below the Ernst angle for an averaged, assumed T1 of muscle tissue. The occurring distortion-effects of a (too) large FA on the slice profiles for a given TR/T1 were analyzed on a homogeneous cylinder-phantom as shown in S8 Fig.

In addition to unavoidable B1 errors corrupting an ideal slice profile in 2D imaging [38], inflow effects may influence the MR signal, especially in larger vessels. Global inversion or saturation pulses are often applied to minimize these effects [14,16], but are at the expense of temporal resolution which was not acceptable for the required parameters and, in particular for segmented sequences, signal homogeneity between excitations may be deteriorated.

The segmented EPI sequence with acceleration by SMS is expected to achieve similar image quality in terms of spatial accuracy compared to a single-shot EPI sequence with PI yielding a similar overall acceleration. The desired TEs restrict echo spacing and phase evolution along the echo train results in distortions, which are likely to appear at a similar level in PI and SMS. Despite spatial accuracy, the SNR penalty in PI remains a clear drawback when compared to SMS. To further reduce scan time in the future, the SMS method used in this work could be combined with standard k-space (under-)sampling strategies such as PI and keyhole methods [17,39]. Because SNR losses in SMS are limited by the g-factor only [17], additional in-plane undersampling could be combined with SMS as long as induced CAIPIRINHA shifts do not negatively interfere. A large number of receiver coils as well as their sophisticated positioning can achieve a well-defined coil sensitivity profile to reduce g-factor limitations which are of high importance for these types of reconstruction.

Moreover, segmentation of k-space data could allow retrospective acceleration. The acquisition time of the individual segments distributes equally over the volume (looping scheme in Figs 1 and 2), which results in temporally discrete EPI segments. Temporal resolution could be improved by 25% for the parameters used in this study by omitting every other EPI segment and performing a PI reconstruction.

A sliding-window (SW) approach could be another reconstruction strategy where the most recently acquired segments update the oldest ones [40]. In the segmented EPI acquisition scheme a portion of low and high spatial frequencies, reflecting global CA enhancement and local details, is acquired in each segment.

## Conclusion

In conclusion, it has been demonstrated that the incorporation of SMS into multi-contrast, segmented EPI can overcome existing limitations in dynamic MR imaging by using a T1 and T2* separation procedure. The technique retains the temporal and spatial resolution even for relatively large FOVs that encompass a feeding artery for AIF detection. SMS is beneficial to in-plane acceleration (PI) because SNR is only reduced by the g-factor and not by the acceleration factor √R. The possible impact of the varying image contrast on the SG algorithm was evaluated. Based on these investigations, it was shown that a constant SG kernel avoids contrast dependencies in SMS image reconstruction, and allows dynamic data to be successfully separated into individual signal contributions that provide additional information for comprehensive pharmacokinetic modelling.

## Supporting information

S1 Fig Simulation of MR signal for different parameter settings to estimate the resulting image contrast. ACS with FA = 5° (blue) and ACS with FA = 90° were used to calculate SG/SSG kernels and to reconstruct image data (FA = 5°) of a phantom containing differently CA-doped sub-volumes. All other imaging parameters were kept constant, i.e. TR = 30 ms.
(TIF)

S2 Fig Subject #2. Dynamics of the measured signal (TE1, TE2, TE3) (top left) and separated signal components (S_0_, T_2_*) (bottom left) during the CA bolus phase in a single voxel (AIF ROI). In contrast to the AIF the extravasation process into muscle tissue is selected from a larger ROI (tissue ROI) removing underestimation of the dynamic signal (TE = 0 ms) due to T_2_*-effects if compared to the directly measured signals (TE1, TE2, TE3).
(TIF)

S3 Fig Subject #3. Dynamics of the measured signal (TE1, TE2, TE3) (top left) and separated signal components (S_0_, T_2_*) (bottom left) during the CA bolus phase in a single voxel (AIF ROI). In contrast to the AIF the extravasation process into muscle tissue is selected from a larger ROI (tissue ROI) removing underestimation of the dynamic signal (TE = 0 ms) due to T_2_*-effects if compared to the directly measured signals (TE1, TE2, TE3).
(TIF)

S4 Fig Subject #4. Dynamics of the measured signal (TE1, TE2, TE3) (top left) and separated signal components (S_0_, T_2_*) (bottom left) during the CA bolus phase in a single voxel (AIF ROI). In contrast to the AIF the extravasation process into muscle tissue is selected from a larger ROI (tissue ROI) removing underestimation of the dynamic signal (TE = 0 ms) due to T_2_*-effects if compared to the directly measured signals (TE1, TE2, TE3).
(TIF)

S5 Fig Subject #2. Evaluation of different SMS reconstruction approaches. Four representative slices from one MB slice group are shown as maps for the normalized subtraction error compared to a single-band acquisition (top) and for the g-factor resulting from the SMS reconstruction (bottom). Histograms below each map summarize the respective metric across all 24 slices. Mean and standard deviation are displayed in the top corner of the histograms.
(TIF)

S6 Fig Subject #3. Evaluation of different SMS reconstruction approaches. Four representative slices from one MB slice group are shown as maps for the normalized subtraction error compared to a single-band acquisition (top) and for the g-factor resulting from the SMS reconstruction (bottom). Histograms below each map summarize the respective metric across all 24 slices. Mean and standard deviation are displayed in the top corner of the histograms.
(TIF)

S7 Fig Subject #4. Evaluation of different SMS reconstruction approaches. Four representative slices from one MB slice group are shown as maps for the normalized subtraction error compared to a single-band acquisition (top) and for the g-factor resulting from the SMS reconstruction (bottom). Histograms below each map summarize the respective metric across all 24 slices. Mean and standard deviation are displayed in the top corner of the histograms.
(TIF)

S8 Fig Comparison of slice profiles at the identical spatial locations resulting from two separate single-band excitations (dashed) versus a multiband pulse (MB = 2; solid). The dependency of slice profiles of the FA for a given TR/T1 is demonstrated. Slice profiles were acquired in a cylindrical phantom (T1 = 106 ms) for a ratio of TR/T1 = 0.3 which is similar to the experimental configuration with an expected, averaged T1 = 870 ms (muscle tissue at 1.5 T [R1]) and used TR = 261 ms. FA above the Ernst angle (α_E_ = 42°) result in severe B1 errors and therefore discrepancies between the ideal and the real slice-profile [38]. Slice profiles are normalized to their maximum signal. R1. Matt A. Bernstein, Ph.D., Kevin F. King, Ph.D., and Xiaohong Joe Zhou PD. Handbook of MRI Pulse Sequences.
(TIF)

S9 Fig Slice profiles for 24 slices of 5 mm thickness (1 mm gap) after MB excitations with *MB* = 4 (solid) compared to the corresponding slice profiles after SB excitations (dashed) (a). Data was taken in homogeneous cylinder-phantom (T1 = 106 ms) where full longitudinal relaxation was guaranteed (TR = 1800 ms), such that the signal levels for both excitations were nearly identical. The zoomed view of one MB slice group in (b) shows the undesired off-resonance signal that originates from imperfections of RF power amplifier hardware and which will interfere with adjacent slices from other MB excitations in case of relatively short TR.
(TIF)

S10 Fig Acquisition of 24 slices in a compound phantom (structure-phantom and bottle-phantom) as shown in (a). Images of SB excitation are given as reference. The slice thickness was set to 5 mm (1 mm gap). The colored difference maps in (b) and (c) show the SB images which were compared to SG/SSG reconstructions after MB excitation (MB = 4, CAIPIRINHA shift of FOV/4). The proposed SG reconstruction method with separately measured ACS source and ACS target data exhibits slice leakage from other simultaneously excited slices of the same MB slice group (b), but results in more correct recovery of the signal intensity and image contrast as the SSG reconstruction (c), where only SB ACS data were considered for the calculation of the reconstruction weights. No masking was applied to keep leakage-signal of CAIPIRINHA shifted slices.
(TIF)

S1 Table The robustness of the reconstruction with respect to S_0_ (top rows) and T_2_* (bottom rows) were quantified by a linear model (b_0_, b_1_). Slice-GRAPPA (left) and split slice-GRAPPA (right) were compared as well as different SNR-levels (1, 5 and 10 avg.) and combinations of echo times to derive the ACS from (ACS111, ACS123). The signal differences are depicted in detail in Figs 7 and 8.
(TIF)

S1 Text Imperfections and their effects in the MB RF-pulse. (DOCX)
