# Supplementary figures and images for: Simultaneous multislice acquisition with multi-contrast segmented EPI for separation of signal contributions in dynamic contrast-enhanced imaging
Source: PLoS One. 2018 Aug 28;13(8):e0202673. doi: 10.1371/journal.pone.0202673 (PMC6112664; doi:10.1371/journal.pone.0202673)

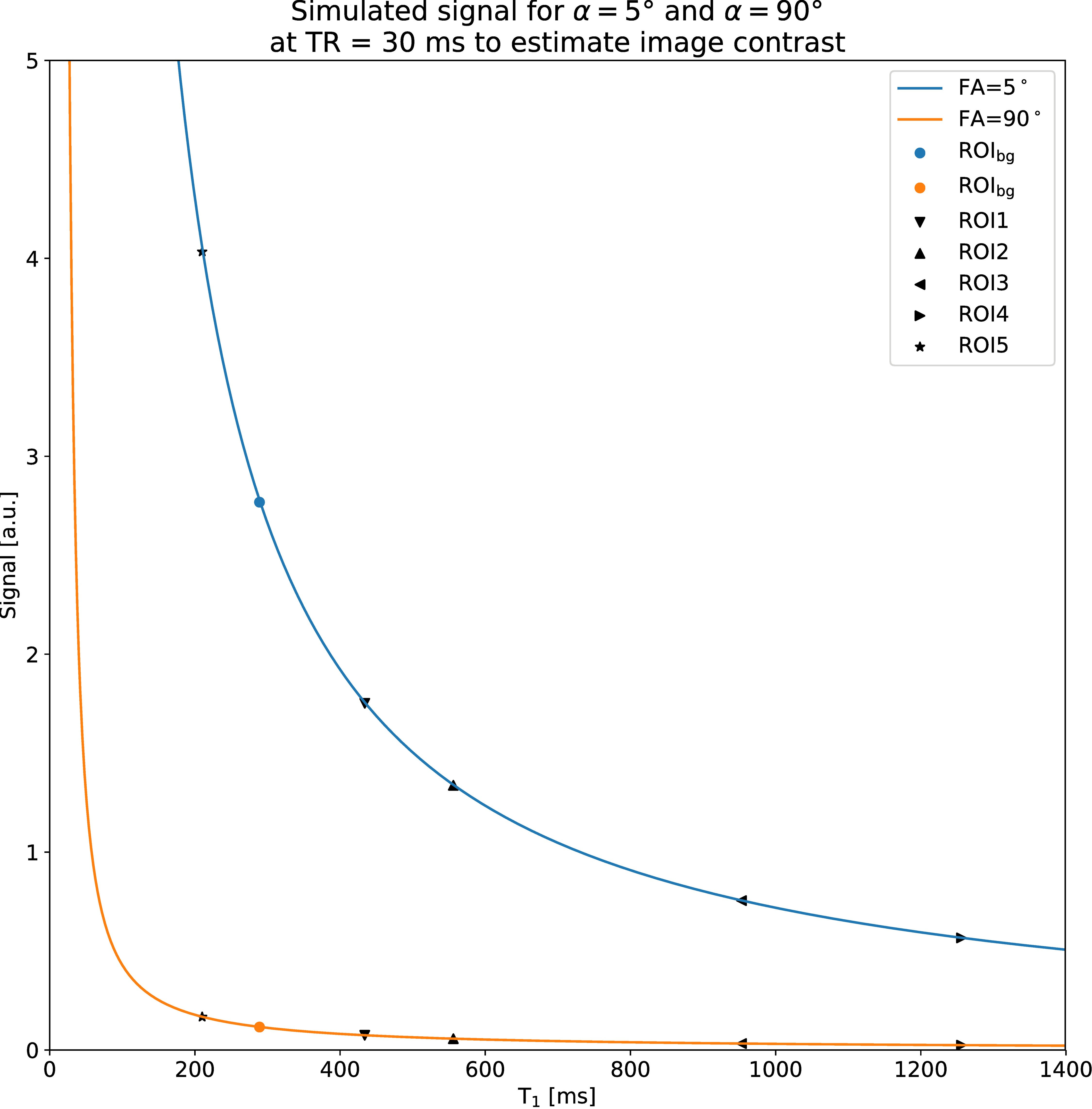

Supplement: S1 Fig — ACS with FA = 5° (blue) and ACS with FA = 90° were used to calculate SG/SSG kernels and to reconstruct image data (FA = 5°) of a phantom containing differently CA-doped sub-volumes. All other imaging parameters were kept constant, i.e. TR = 30 ms. (TIF) [file pone.0202673.s001.tif]

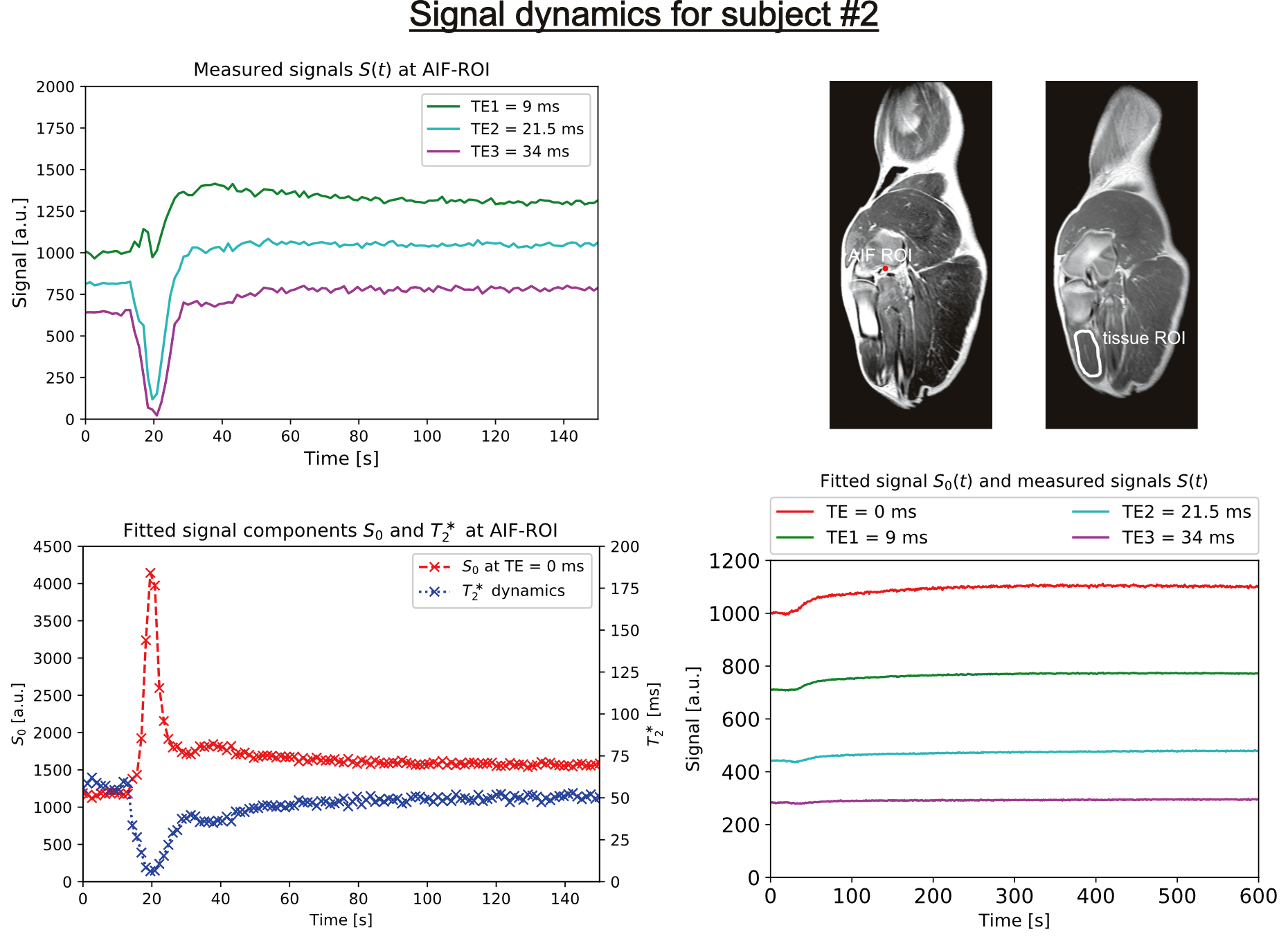

Supplement: S2 Fig — Dynamics of the measured signal (TE1, TE2, TE3) (top left) and separated signal components (S0, T2*) (bottom left) during the CA bolus phase in a single voxel (AIF ROI). In contrast to the AIF the extravasation process into muscle tissue is selected from a larger ROI (tissue ROI) removing underestimation of the dynamic signal (TE = 0 ms) due to T2*-effects if compared to the directly measured signals (TE1, TE2, TE3). (TIF) [file pone.0202673.s002.tif]

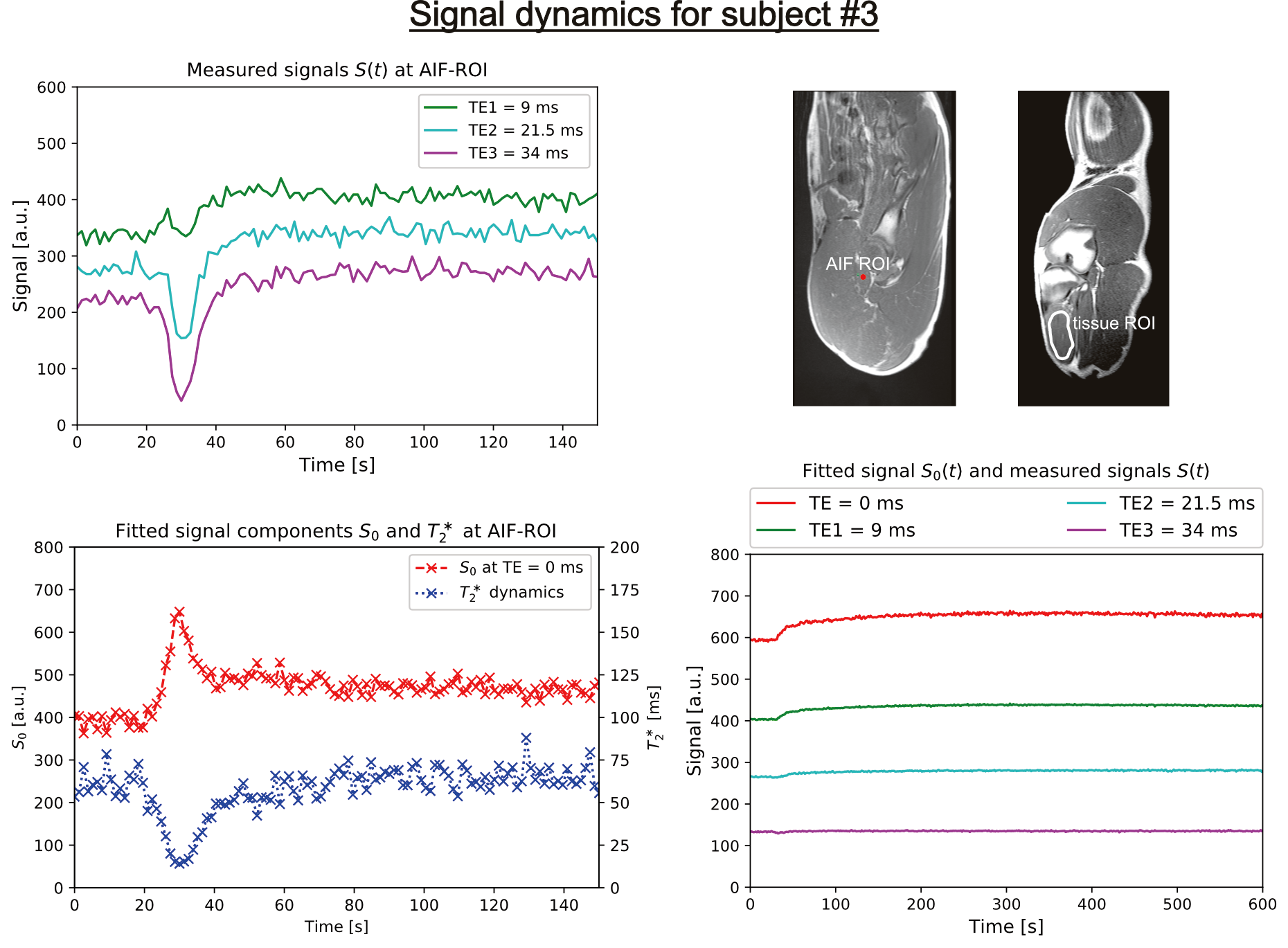

Supplement: S3 Fig — Dynamics of the measured signal (TE1, TE2, TE3) (top left) and separated signal components (S0, T2*) (bottom left) during the CA bolus phase in a single voxel (AIF ROI). In contrast to the AIF the extravasation process into muscle tissue is selected from a larger ROI (tissue ROI) removing underestimation of the dynamic signal (TE = 0 ms) due to T2*-effects if compared to the directly measured signals (TE1, TE2, TE3). (TIF) [file pone.0202673.s003.tif]

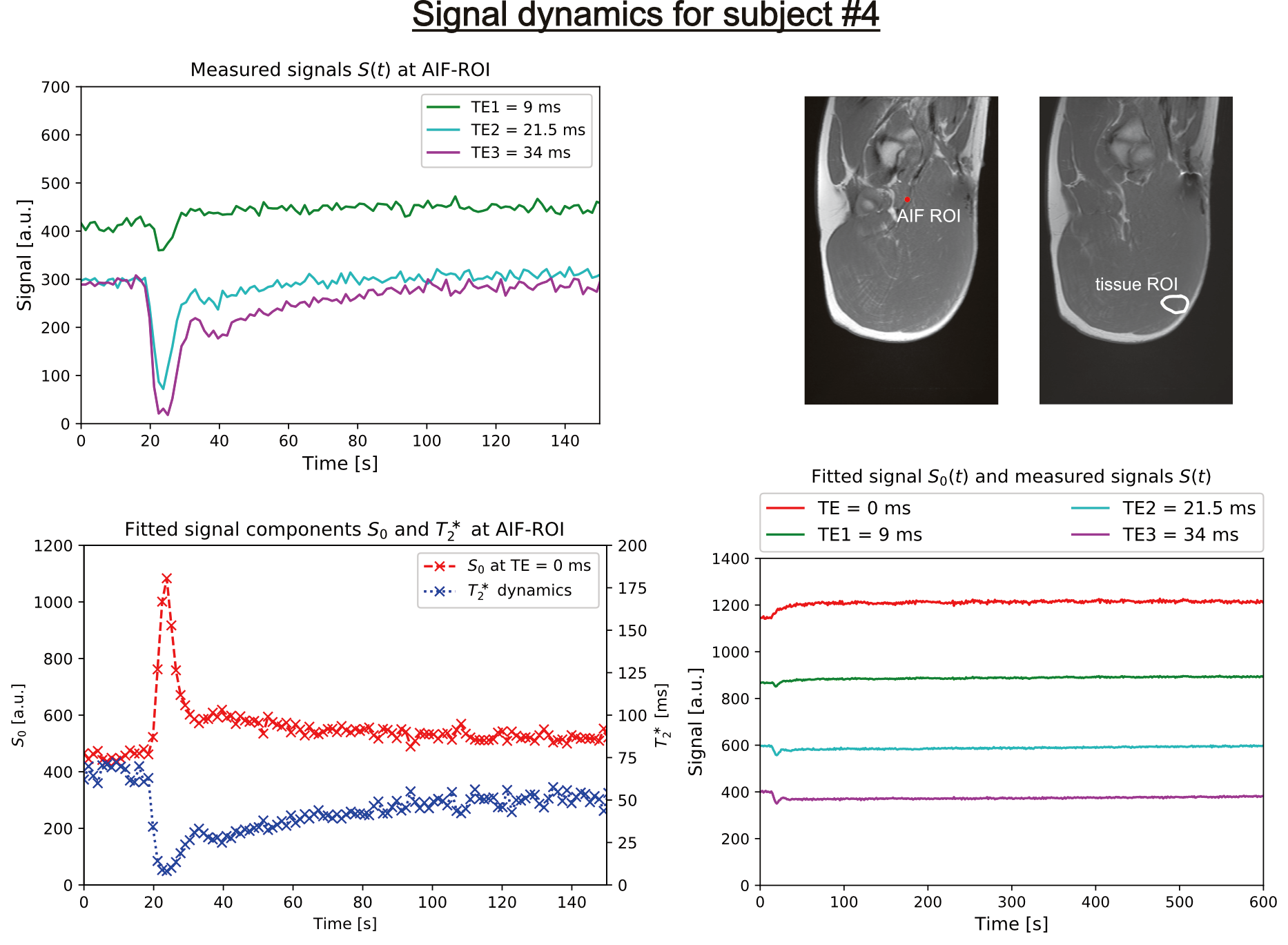

Supplement: S4 Fig — Dynamics of the measured signal (TE1, TE2, TE3) (top left) and separated signal components (S0, T2*) (bottom left) during the CA bolus phase in a single voxel (AIF ROI). In contrast to the AIF the extravasation process into muscle tissue is selected from a larger ROI (tissue ROI) removing underestimation of the dynamic signal (TE = 0 ms) due to T2*-effects if compared to the directly measured signals (TE1, TE2, TE3). (TIF) [file pone.0202673.s004.tif]

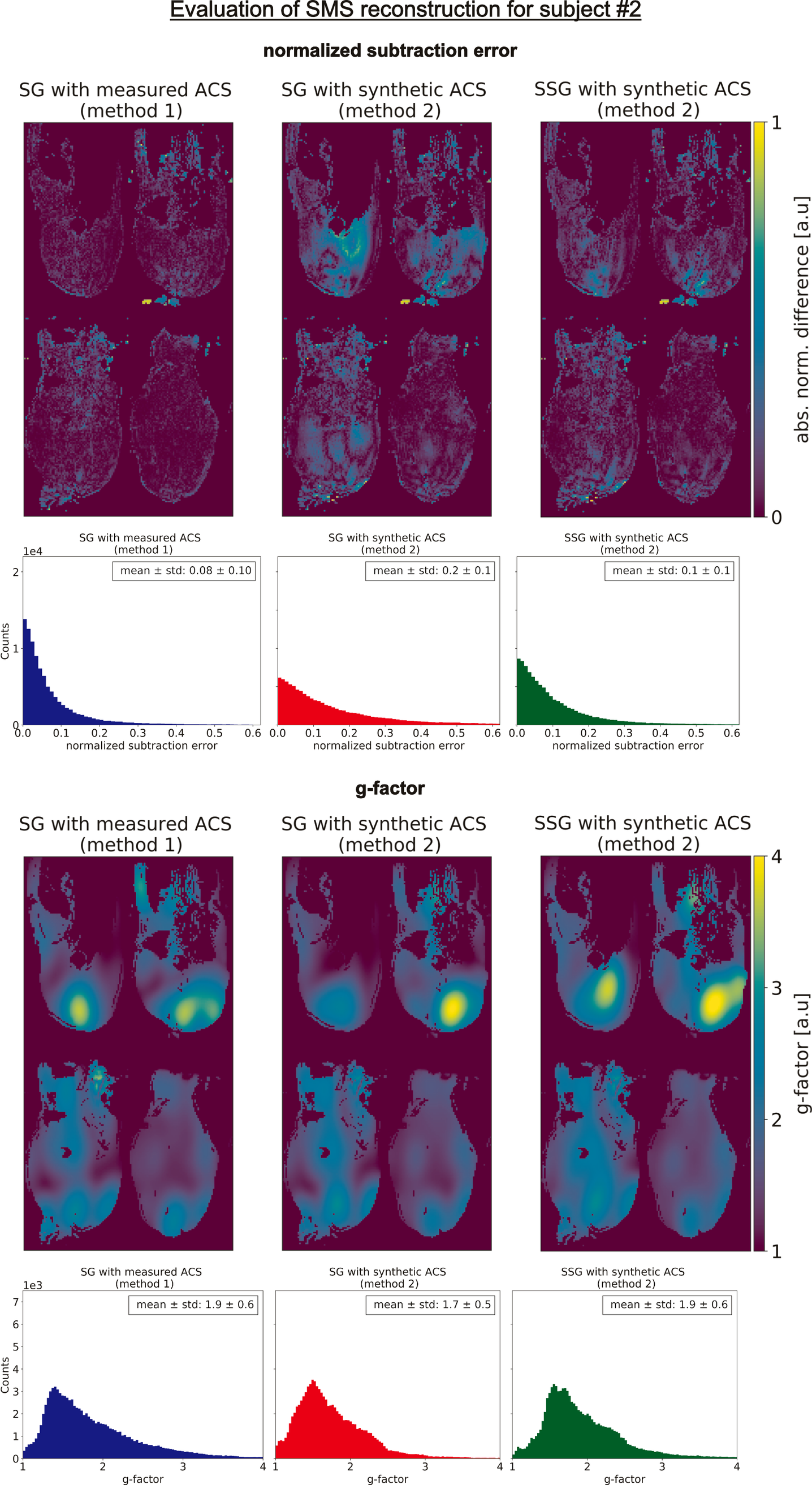

Supplement: S5 Fig — Evaluation of different SMS reconstruction approaches. Four representative slices from one MB slice group are shown as maps for the normalized subtraction error compared to a single-band acquisition (top) and for the g-factor resulting from the SMS reconstruction (bottom). Histograms below each map summarize the respective metric across all 24 slices. Mean and standard deviation are displayed in the top corner of the histograms. (TIF) [file pone.0202673.s005.tif]

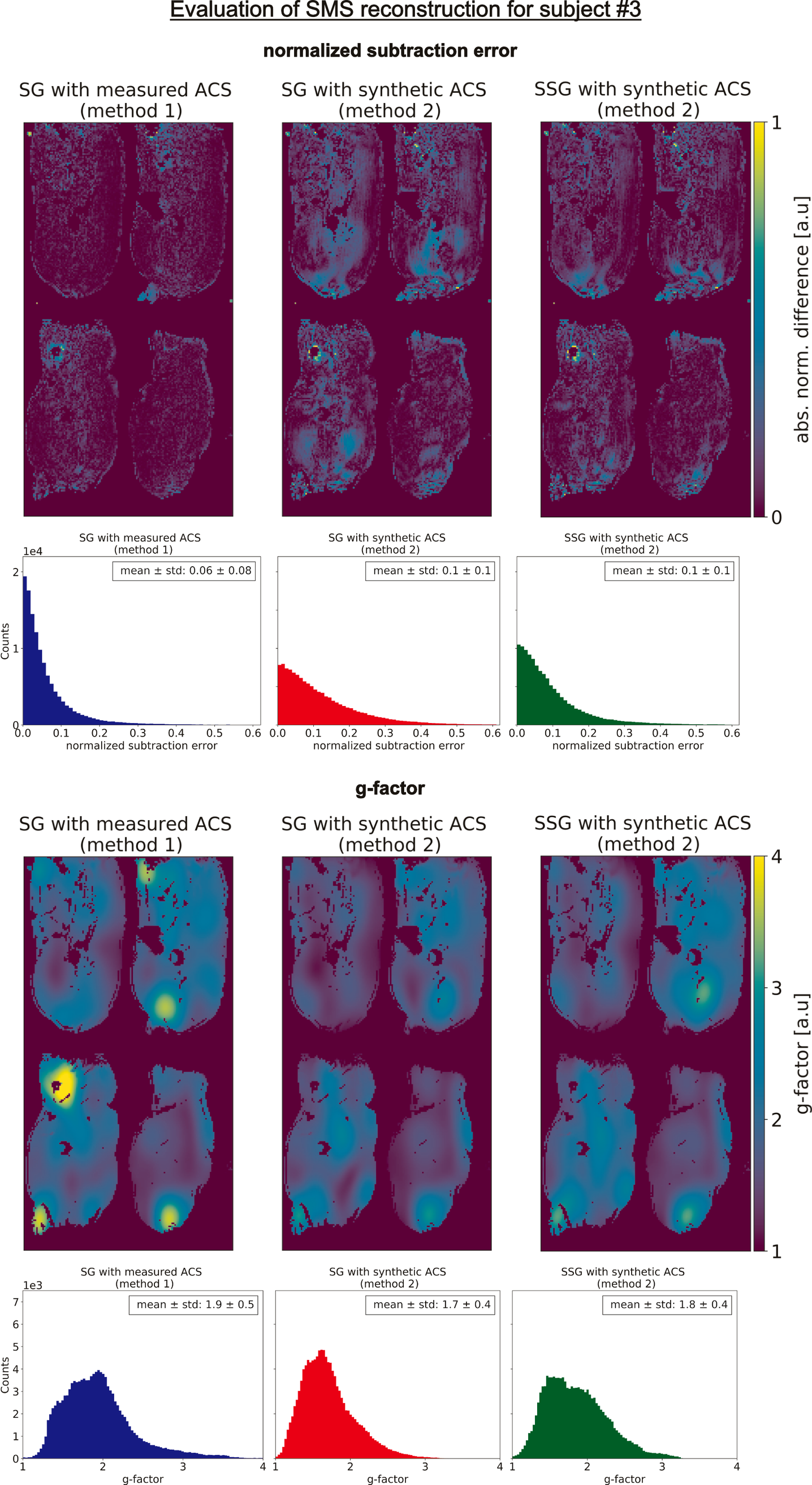

Supplement: S6 Fig — Evaluation of different SMS reconstruction approaches. Four representative slices from one MB slice group are shown as maps for the normalized subtraction error compared to a single-band acquisition (top) and for the g-factor resulting from the SMS reconstruction (bottom). Histograms below each map summarize the respective metric across all 24 slices. Mean and standard deviation are displayed in the top corner of the histograms. (TIF) [file pone.0202673.s006.tif]

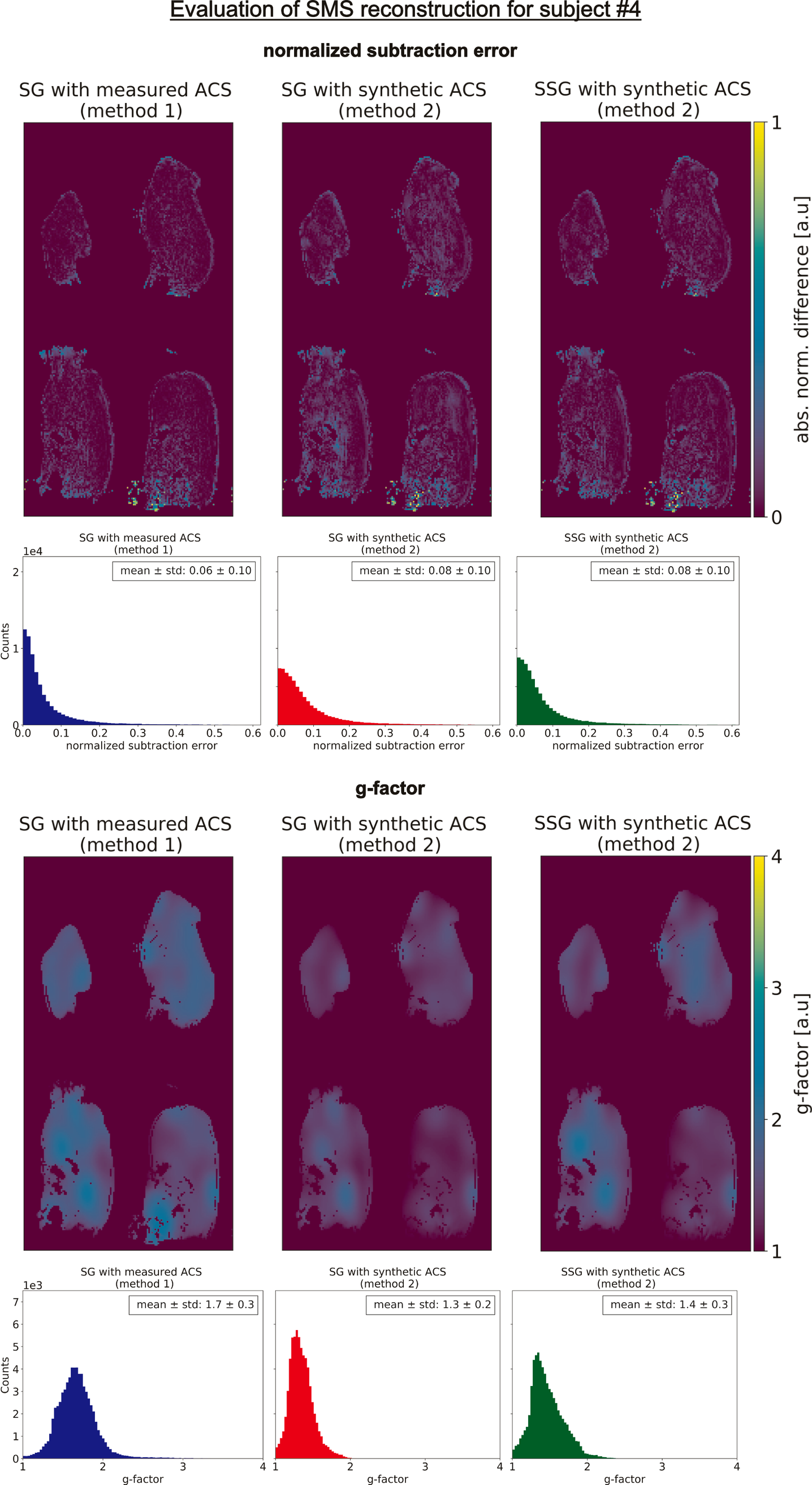

Supplement: S7 Fig — Evaluation of different SMS reconstruction approaches. Four representative slices from one MB slice group are shown as maps for the normalized subtraction error compared to a single-band acquisition (top) and for the g-factor resulting from the SMS reconstruction (bottom). Histograms below each map summarize the respective metric across all 24 slices. Mean and standard deviation are displayed in the top corner of the histograms. (TIF) [file pone.0202673.s007.tif]

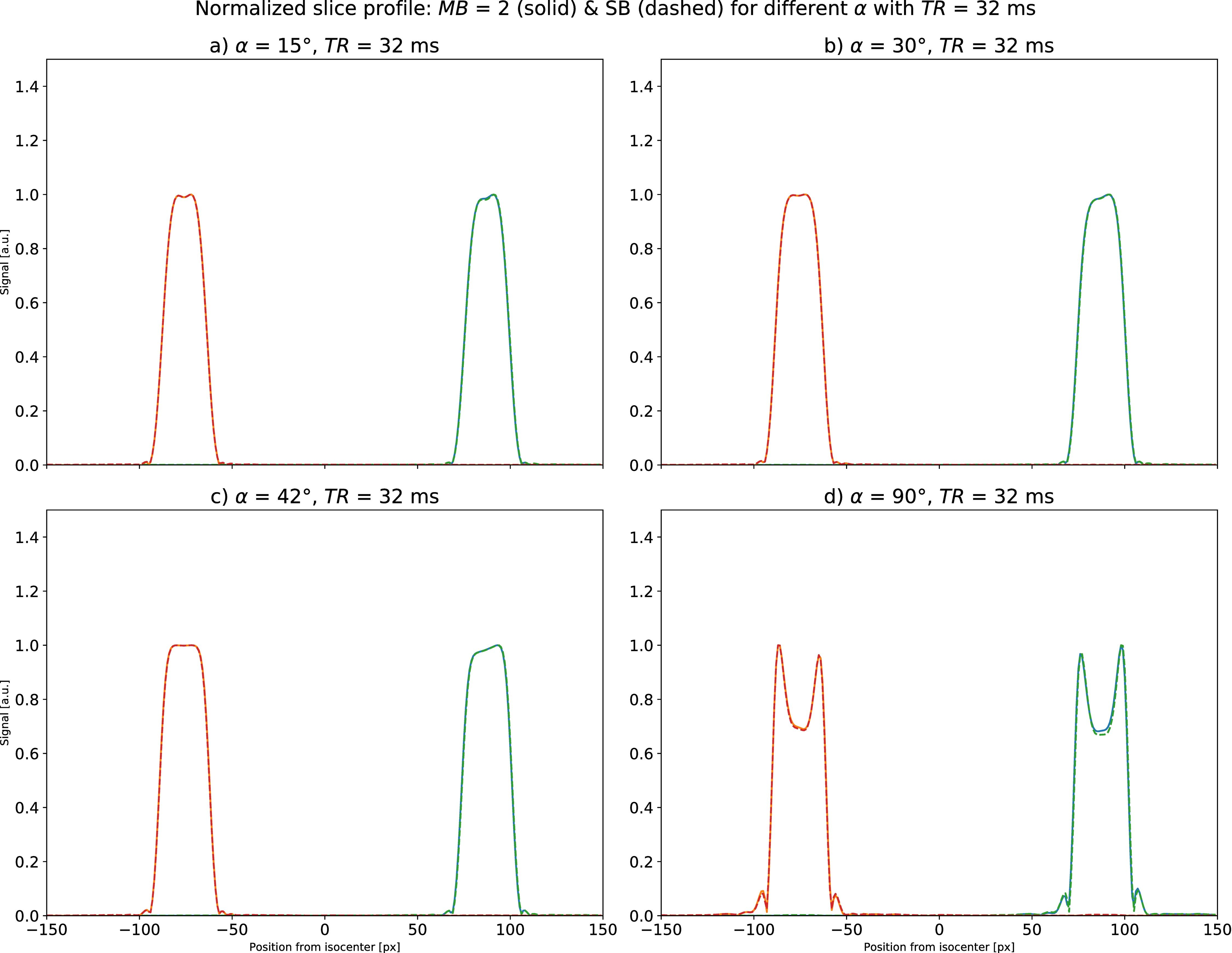

Supplement: S8 Fig — The dependency of slice profiles of the FA for a given TR/T1 is demonstrated. Slice profiles were acquired in a cylindrical phantom (T1 = 106 ms) for a ratio of TR/T1 = 0.3 which is similar to the experimental configuration with an expected, averaged T1 = 870 ms (muscle tissue at 1.5 T [R1]) and used TR = 261 ms. FA above the Ernst angle (αE = 42°) result in severe B1 errors and therefore discrepancies between the ideal and the real slice-profile [38]. Slice profiles are normalized to their maximum signal. R1. Matt A. Bernstein, Ph.D., Kevin F. King, Ph.D., and Xiaohong Joe Zhou PD. Handbook of MRI Pulse Sequences. (TIF) [file pone.0202673.s008.tif]

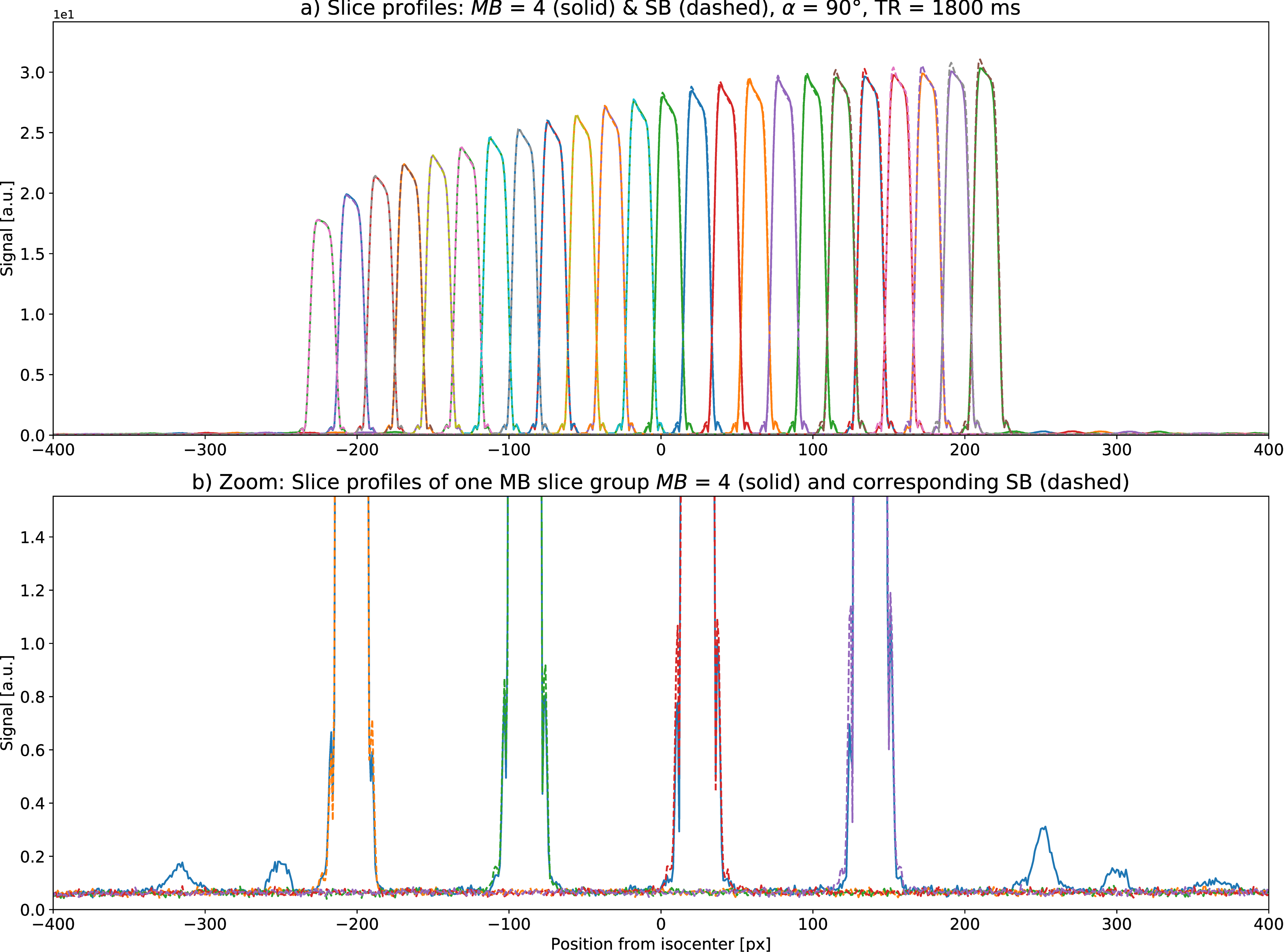

Supplement: S9 Fig — Slice profiles for 24 slices of 5 mm thickness (1 mm gap) after MB excitations with MB = 4 (solid) compared to the corresponding slice profiles after SB excitations (dashed) (a). Data was taken in homogeneous cylinder-phantom (T1 = 106 ms) where full longitudinal relaxation was guaranteed (TR = 1800 ms), such that the signal levels for both excitations were nearly identical. The zoomed view of one MB slice group in (b) shows the undesired off-resonance signal that originates from imperfections of RF power amplifier hardware and which will interfere with adjacent slices from other MB excitations in case of relatively short TR. (TIF) [file pone.0202673.s009.tif]

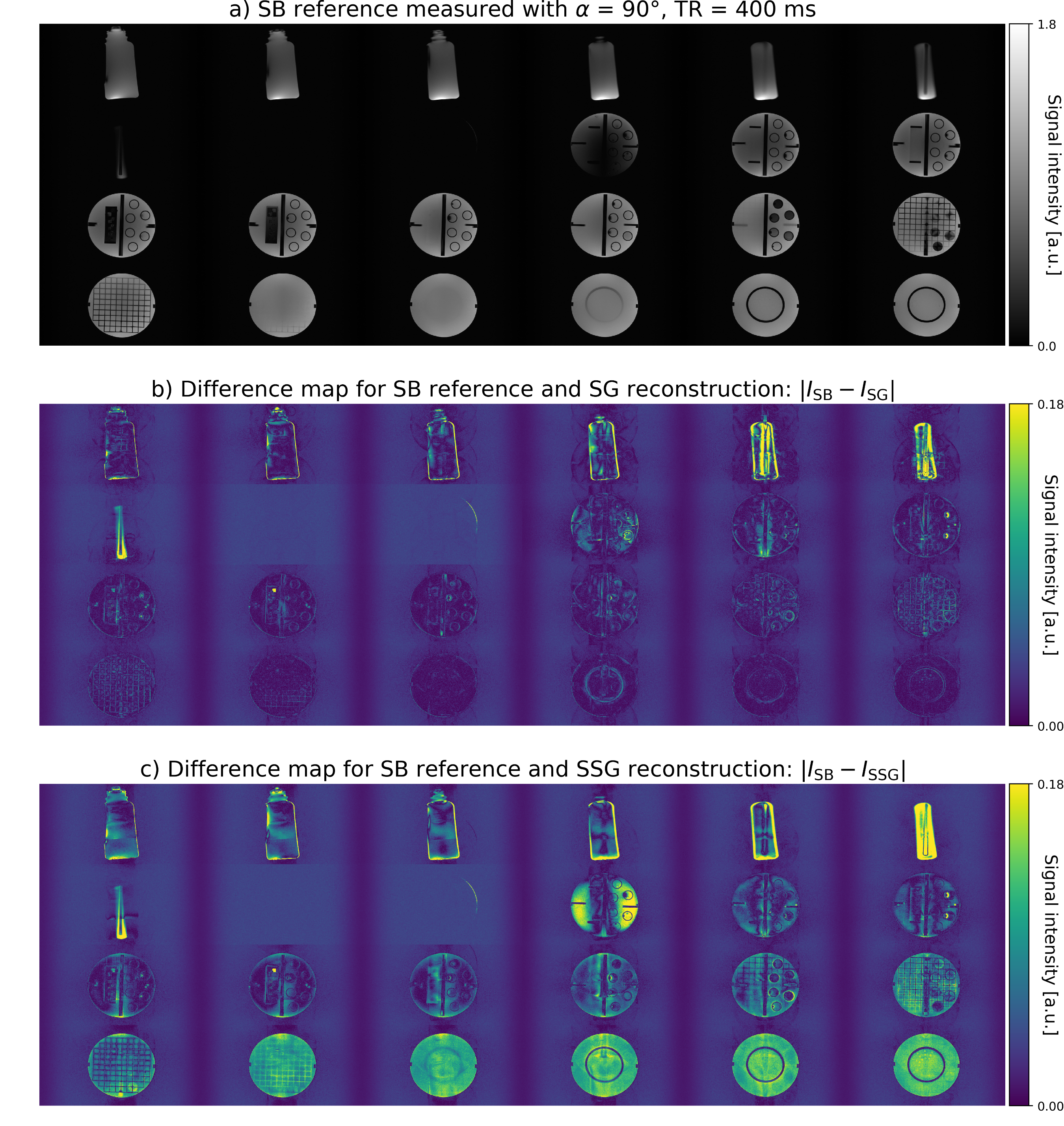

Supplement: S10 Fig — Acquisition of 24 slices in a compound phantom (structure-phantom and bottle-phantom) as shown in (a). Images of SB excitation are given as reference. The slice thickness was set to 5 mm (1 mm gap). The colored difference maps in (b) and (c) show the SB images which were compared to SG/SSG reconstructions after MB excitation (MB = 4, CAIPIRINHA shift of FOV/4). The proposed SG reconstruction method with separately measured ACS source and ACS target data exhibits slice leakage from other simultaneously excited slices of the same MB slice group (b), but results in more correct recovery of the signal intensity and image contrast as the SSG reconstruction (c), where only SB ACS data were considered for the calculation of the reconstruction weights. No masking was applied to keep leakage-signal of CAIPIRINHA shifted slices. (TIF) [file pone.0202673.s010.tif]

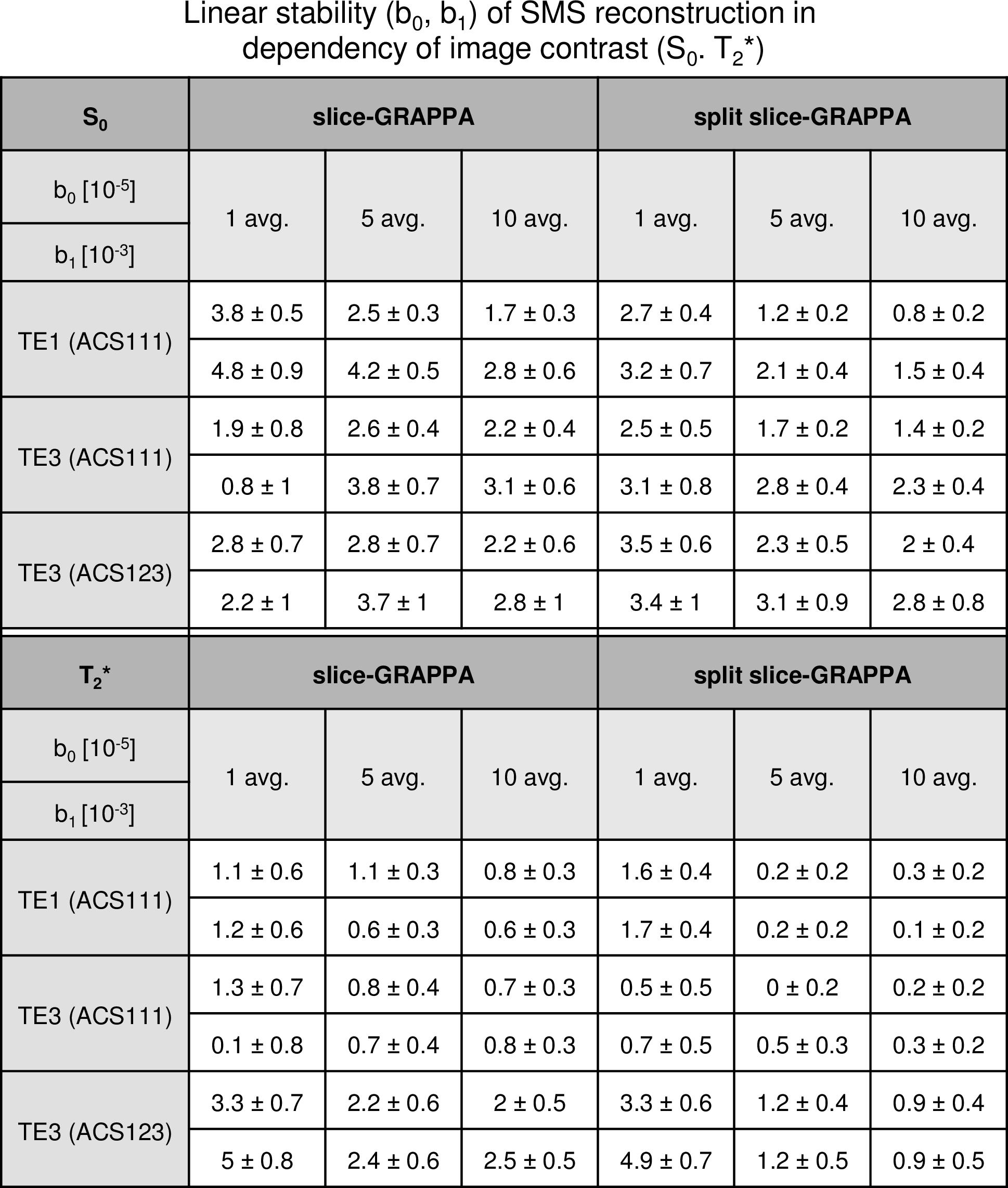

Supplement: S1 Table — Slice-GRAPPA (left) and split slice-GRAPPA (right) were compared as well as different SNR-levels (1, 5 and 10 avg.) and combinations of echo times to derive the ACS from (ACS111, ACS123). The signal differences are depicted in detail in Figs 7 and 8. (TIF) [file pone.0202673.s011.tif]
