# Supplementary material for: Simultaneous multislice acquisition with multi-contrast segmented EPI for separation of signal contributions in dynamic contrast-enhanced imaging
Source: PLoS One. 2018 Aug 28;13(8):e0202673. doi: 10.1371/journal.pone.0202673 (PMC6112664; doi:10.1371/journal.pone.0202673)
Supplement: S1 Text — (DOCX) [file pone.0202673.s012.docx]

S1 Text: Two different methods for the formation of the ACS were evaluated as described previously. Measured ACS source and ACS target data can be considered for the calculation of the reconstruction weights in SG, whereas the SSG algorithm processes ACS data from a SB acquisition. In standard approaches, the ACS data acquired with SB excitations are either synthesized into a MB dataset as ACS source (SG) or kept separated and zero-filled (SSG) [17,26]. However, if the MB RF-pulse is calculated in a straightforward manner as defined in Eq 1, RF power amplifier’s non-linearity and memory effects will distort the real MB RF-pulse which is transmitted [R2]. In particular, the rapidly-varying envelopes for excitations with higher MB-factors experience these limitations [R3]. Different optimizations in RF-pulse design have been proposed to tackle hardware related imperfections, but they usually come at a cost of flexibility and generality [R4].
The transmitted MB RF-pulse was analyzed experimentally in a homogeneous cylinder-phantom (T1 = 106 ms). The phantom was positioned at isocenter and the readout was aligned along slice direction to sample the profiles of the excited slices. If MB excitations are performed as described in Eq 1, inter-slice artifacts at off-resonance positions will occur as shown in S9 Fig and as mentioned in [R4]. These can cause interference with neighboring slices of other MB slice groups. The severity of these effects depends on the system’s hardware and protocol parameters, e.g. MB-factor, FA, TR and spacing of slices within one MB slice group [R4].
The effects of a distorted MB RF-pulse were investigated on a compound phantom made up of a structure- and a bottle-phantom. 24 slices (5 mm thickness, 1 mm gap) of MRI MB data were acquired with *MB* = 4 and a CAIPIRINHA shift of FOV/4. These were reconstructed with/without measured MB ACS source data according to the two presented methods for SG and SSG. S10 Fig shows the comparison of the reconstruction results to reference images acquired with SB (a). By taking imperfections of the transmitted MB RF-pulse into account when calculating the reconstruction weights, the level of slice specific distortions can be reduced and the recovered signal intensity matches the SB reference more correctly (b) as if ACS are delivered by a SB acquisition only (c). Reductions in TR, which are essential in SMS, will emphasize these effects because interference of off-resonance excitations with adjacent slices of other MB slice groups may increase.
However, depending on the spatial configuration of the receiver coils and the distance between the slices in one MB slice group, these variations caused by the MB RF-pulse have to be traded against inter-slice leakage which can be reliably blocked by SSG [26].
In addition, the acquisition of separate MB ACS source and SB ACS target data is prone to any motion and might therefore result in a mismatch between ACS source and ACS target if motion can not be controlled sufficiently.

R2. Scott GC. MRI Transmitter Amplifier Systems. Proc Intl Soc Mag Reson Med. 2012;

R3. Grissom WA, Kerr AB, Stang P, Scott GC, Pauly JM. Minimum envelope roughness pulse design for reduced amplifier distortion in parallel excitation. Magn Reson Med. 2010;64: 1432–1439. doi:10.1002/mrm.22512

R4. Abo Seada S, Price AN, Hajnal J V., Malik SJ. Optimized amplitude modulated multiband RF pulse design. Magn Reson Med. 2017;78: 2185–2193. doi:10.1002/mrm.26610
